# A transcriptional blueprint for a spiral-cleaving embryo

**DOI:** 10.1186/s12864-016-2860-6

**Published:** 2016-08-05

**Authors:** Hsien-Chao Chou, Margaret M. Pruitt, Benjamin R. Bastin, Stephan Q. Schneider

**Affiliations:** 1 Department of Genetics, Development and Cell Biology, Iowa State University, 503 Science Hall II, Ames, IA 50011 USA; 2 Present Address: National Cancer Institute, US National Institutes of Health, Bethesda, Maryland USA; 3 Present Address: Department of Pediatrics, University of Chicago, Chicago, IL USA

**Keywords:** Spiral cleavage, Lophotrochozoan, Annelid, Transcriptome, Maternal contribution, Zygotic transcription

## Abstract

**Background:**

The spiral cleavage mode of early development is utilized in over one-third of all animal phyla and generates embryonic cells of different size, position, and fate through a conserved set of stereotypic and invariant asymmetric cell divisions. Despite the widespread use of spiral cleavage, regulatory and molecular features for any spiral-cleaving embryo are largely uncharted. To address this gap we use RNA-sequencing on the spiralian model *Platynereis dumerilii* to capture and quantify the first complete genome-wide transcriptional landscape of early spiral cleavage.

**Results:**

RNA-sequencing datasets from seven stages in early *Platynereis* development, from the zygote to the protrochophore, are described here including the *de novo* assembly and annotation of ~17,200 *Platynereis* genes. Depth and quality of the RNA-sequencing datasets allow the identification of the temporal onset and level of transcription for each annotated gene, even if the expression is restricted to a single cell. Over 4000 transcripts are maternally contributed and cleared by the end of the early spiral cleavage phase. Small early waves of zygotic expression are followed by major waves of thousands of genes, demarcating the maternal to zygotic transition shortly after the completion of spiral cleavages in this annelid species.

**Conclusions:**

Our comprehensive stage-specific transcriptional analysis of early embryonic stages in *Platynereis* elucidates the regulatory genome during early spiral embryogenesis and defines the maternal to zygotic transition in *Platynereis* embryos. This transcriptome assembly provides the first systems-level view of the transcriptional and regulatory landscape for a spiral-cleaving embryo.

**Electronic supplementary material:**

The online version of this article (doi:10.1186/s12864-016-2860-6) contains supplementary material, which is available to authorized users.

## Background

**Fig. 1:**
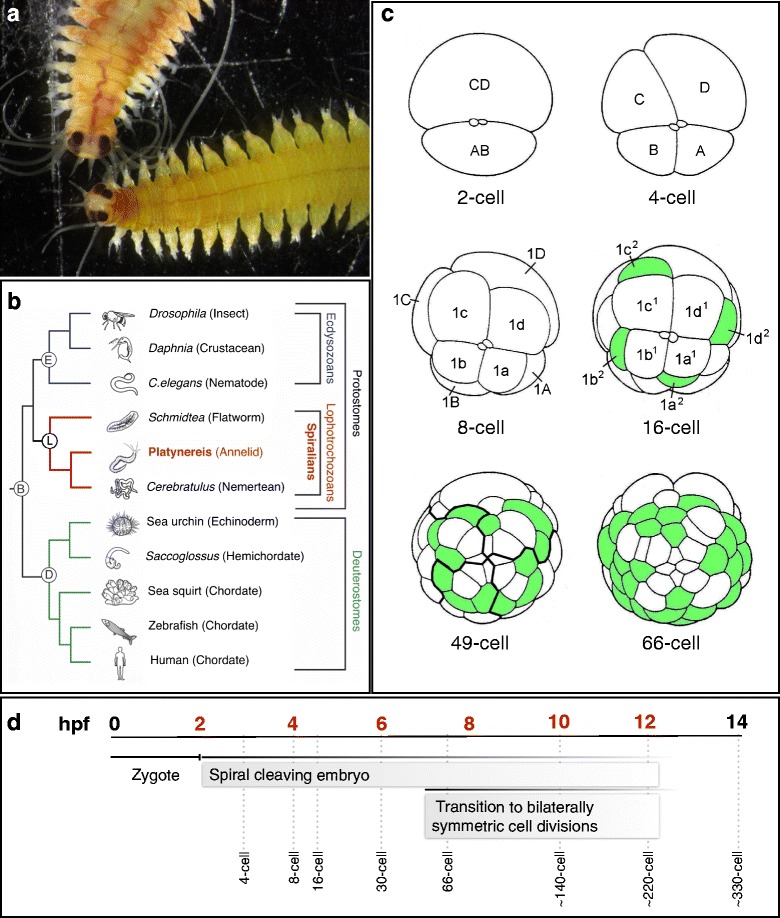
Early development of the marine annelid *Platynereis dumerilii.* **a** Dorsal views of *Platynereis* adults showing the head and anterior segments of a male (top) and female (bottom). **b** Phylogenetic position of the spiralian annelid *Platynereis dumerilii* (highlighted in red). Bilaterally symmetric animals comprise of deuterostomes and protostomes. Within protostomes there are two clades, ecdysozoans and lophotrochozoans. Many lophotrochozoan phyla are spiralians. **c** Early spiral cleavage patterns in *Platynereis*. The first cleavage is highly asymmetric, giving rise to the AB cell and the much larger CD cell (2-cell). The second cleavage creates the macromeres **a**, **b**, **c** and **d** (4-cell). The third cleavage (8-cell) is the first cleavage along the animal/vegetal axis and marks the beginning of spiral cleavage. The animal-pole micromeres, denoted with lowercase letters, are shifted slightly clockwise with respect to their macromere sisters, denoted with uppercase letters. At the fourth cleavage (16-cell), animal-pole daughter cells are shifted counter-clockwise compared to their more vegetal sister cells. This spiral pattern of cell divisions continues throughout early embryogenesis and transitions in some cells to bilaterally symmetric cell divisions (66-cell). Cells depicted in green will form multi-ciliated cells by 12 h of development. The four earlier stages show two small polar bodies at the animal pole. **d** The developmental timeline of *Platynereis dumerilii* from the zygote to the protrochophore. The first cleavage occurs around 2 h post fertilization (hpf) and the spiral cleavage pattern (described in **c**) continues throughout the first ~12 h of development. *Platynereis* transitions to a pattern of bilaterally symmetric cell divisions around 7hpf. The dashed lines show the approximate ages of various cell stages. The time points at which samples were collected for RNA-sequencing are in red

Over one-third of all animal phyla belong to the clade Spiralia, which includes annelids like earthworms and leeches, mollusks like snails and clams, flatworms like planarians, and many other smaller, enigmatic phyla [1]. Spiralians comprise one of the three major radiations of bilaterian animals, grouped as lophotrochozoans (Fig. 1b) that originated during the Precambrium utilizing a common mode of early embryogenesis called spiral cleavage [2–5]. Spiral cleavage refers to a pattern of stereotypic, invariant asymmetric cell divisions that generate cells of different size and fate during early embryogenesis [1]. Most obvious after the 4-cell stage, the orientations of mitotic spindles during subsequent cell divisions are tilted and the orientations of the spindles alternate in regard to their positions along the animal-vegetal axis of the embryo, generating daughter cells that assume a spiral arrangement. Each embryonic cell is defined by its position and birth order, and exhibits a distinct cell fate. These patterns, birth orders, and cell fates of embryonic cells are not only invariant within one species, but are also conserved between species of the same phylum and even among species of several spiralian phyla including annelids and mollusks. Thus, this mode of development enables comparisons between individual embryonic founder cells from different phyla, tracing the common origin of individual cells back to the Precambrian age.

Over the last decade the spiralian *Platynereis dumerilii* has emerged as an excellent model organism for the study of development, evolution and marine biology (Fig. 1a) [6, 7]. One of the main advantages of this marine annelid is that its body plan and genome has maintained many ancient features [8, 9]. For example, the *Platynereis* genome has retained ancestral complements for gene families and gene structure, like the *wnt* family, with 12 of 13 ancient *wnt* genes conserved in *Platynereis* [10, 11]. Furthermore, another comparative study analyzing exon-intron structure shows that *Platynereis* genes are more similar to human genes than to genes from insects and nematodes [12]. This suggests more conserved genomic features between this annelid and vertebrates and an increase in evolutionary changes in insect and nematode lineages (Fig. 1b). *Platynereis* also exhibits many common features with vertebrates including similar signatures of developmental gene expression during the formation of the brain, central nervous system and eye development, and a similar neuropeptide complement [13–17], features that were lost or strongly modified in the evolutionarily closer model systems *Caenorhabditis elegans* and *Drosophila melanogaster*. Although more recent molecular analyses have placed nereids, like *Platynereis,* into a more derived phylogenetic position within annelids [18, 19], *Platynereis* has emerged as a prominent model for comparative studies to infer early bilaterian characteristics.

Another important advantage of *Platynereis* is its accessibility and amenability for experimental analyses. The entire life-cycle of *Platynereis* can be recreated under laboratory conditions, and its lunar synchronized mating behavior makes it possible to collect thousands of synchronously developing embryos at distinct embryonic stages [6]. Additionally, several experimental avenues have been pioneered in recent years in *Platynereis* including zygote microinjection, transient and stable transgenesis, and various genome-modifying technologies that allow functional studies [20, 21].

The first 14 h of *Platynereis* development comprises early embryogenesis, from fertilization to an early protrochophore stage (~330 cells) that has hatched from the vitelline membrane formed shortly after fertilization (Fig. 1c and d) [22–24]. At fertilization and triggered by sperm contact, the fertilized egg completes the meiotic divisions and generates two polar bodies at the animal pole before the zygote enters the first mitotic cell division shortly after 2 h post fertilization (hpf). The first two cell divisions are highly unequal giving rise to four large embryonic founder cells of different size called A, B, C, and D (Fig. 1c). The spiral cleavage mode of embryogenesis is mainly confined to the next four rounds of cell divisions. Each of the four founder cells and their progeny exhibit a similar series of asymmetric cell divisions oriented along the animal-vegetal axis of the embryo, generating animal pole and vegetal pole daughter cells. During consecutive divisions, the orientation of mitotic spindles alternate and generate quartets of daughter cells that are positioned clockwise and counterclockwise when viewed from the animal pole, assuming a spiral arrangement. Each embryonic cell can be identified by its size and position, and has been shown to have a distinct cell fate [24, 25]. The embryonic cells begin to transition to a bilaterally symmetric mode of division after 7hpf, though alternating spindle orientations can still be observed. The bilaterally symmetric pattern of cell divisions generates a pattern of similarly sized cells on the left and right side of the embryo. The larger D-quadrant bears special significance in this transition by creating two large cells, the somatoblast named ‘2d^112^’, and the mesentoblast named ‘4d’ [22, 24]. Both cells divide symmetrically and perpendicular to the animal-vegetal axis, segregating two founder cells each whose progeny will form the left and right side of the trunk ectoderm and trunk mesoderm [22, 24–26]. Thus, by 7hpf, the founder cells for each germ layer have been segregated, and after 9hpf, the germ cells segregate from the mesodermal cell lineages [27, 28]. Twenty-four embryonic cells have stopped dividing by 12hpf and begin to differentiate into multi-ciliated cell types forming a ciliated ring, the prototroch. By 14hpf, an early protrochophore stage of ~330 cells, *Platynereis* has hatched and rotates freely [23, 24].

Although the early development of *Platynereis* has been described and some molecular data for early embryonic stages is emerging [11, 23, 29, 30], a comprehensive transcriptome study for early embryonic stages is lacking. Current transcriptome studies are confined to genes of interests or are focused on later stages [31–33]. Comprehensive sequencing and functional annotation of a developmental *Platynereis* transcriptome is the first crucial step for understanding the molecular dynamics during early development, including maternal and zygotic contributions, and the complex regulatory networks that lead to the different cell fates. The advent of next-generation RNA-sequencing technologies allows us to not only reconstruct gene models, but also to obtain the precise expression level of every transcript throughout early development.

Here we describe the first comprehensive transcriptome draft during early development in *Platynereis* using a *de novo* assembly strategy. We performed mRNA deep sequencing of seven different early developmental stages using Illumina HiSeq sequencing with read lengths ~75–100 bp. 273,087 transcripts were assembled and 51,260 of the transcripts have potential protein-coding regions larger than 100 amino acids. The assembled genes were annotated by comparison of various known protein and pathway databases such as Swiss-Prot [34], Pfam [35], Gene Ontology [36], and KEGG [37]. We identify around 1000 to 2520 differentially expressed genes between adjacent stages with a FDR < 0.001, and our analysis allows for the temporal onset and level of transcription to be described for each of 28,500 genes. Importantly, our data analyses identify maternal contributions and several waves of zygotic transcription during and after the completion of spiral cleavages. This *Platynereis* transcriptome provides the first in-depth view of the transcriptional and regulatory landscapes for a spiral-cleaving embryo, generates a molecular platform for comparison with vertebrates and other model organisms to identify conserved core biological processes necessary for early animal embryogenesis, and enables insights into plasticity and evolution of early animal development.

## Results

### *De novo* assembly of the early *Platynereis dumerilii* transcriptome

**Fig. 2:**
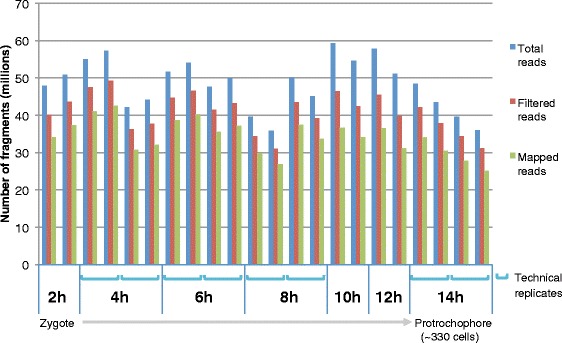
Stage-specific deep sequencing in 2-h intervals throughout early *Platynereis* embryogenesis. RNA-sequencing was performed on seven stages ranging from the zygote (2 h) to the protrochophore (14 h). Each time point sampled includes biological replicates, and there are also technical replicates (blue brackets) for the 4, 6, 8, and 14 h samples. The depth of these libraries range from 35 to 60 million total reads, represented with the blue bars. Filtered reads (red bars) are the total reads after trimming the adaptor sequences and low quality sections of the reads. The filtered reads were used to assemble the transcriptome, and the green bars represent the number of reads mapped back to the assembly. The mapped fragments range from 79 to 91 % of the filtered reads

The unique mating behavior of *Platynereis dumerilii* results in the instantaneous external fertilization of thousands of synchronously developing embryos [6]. The large batches of synchronously developing embryos enables the isolation of sufficient amounts of stage-specific RNA from a single mating for mRNA sequencing libraries without amplification steps. Using this powerful system, we captured the transcriptional activity of genes involved in the early development of *Platynereis* by obtaining mRNA libraries at 2, 4, 6, 8, 10, 12, and 14hpf from the fertilized egg to the ~330-cell stage with biological replicates for each stage (Fig. 1d). Each library was constructed from RNA isolated from one individual batch of developing embryos. Additionally, technical replicates were sequenced from eight samples, covering four stages. The RNA-sequencing libraries each have 35–60 million total reads per sample and 25–40 million mapped reads per sample (Fig. 2).

Since there is no quality reference genome for *Platynereis* available yet, we used the software Trinity [38] to assemble the RNA-sequencing libraries in a genome-independent manner into transcripts. To create unified gene models, we combined all biological replicates together (~785 million paired-end reads), and assembled them in a single pass into 273,087 non-redundant transcripts belonging to 193,310 genes (N50 size: 1466 bp) (see Methods for details). We focused our subsequent analyses on sequences with potential open reading frames (ORFs) larger than 100aa (51,260 transcripts corresponding to 32,257 genes; N50: 3230 bp; N90: 1011 bp). Similar to results from previous *de novo* assemblies [38–40], the vast majority of the remaining 221,826 transcripts consist of shorter sequences (N50: 528 bp; N90: 235 bp) with 93 % not expressed at any stage (FPKM (fragments per kilobase per million mapped reads) < 1: 205,693 transcripts). To evaluate the quality of our transcriptome assembly without a reference genome or transcriptome, we aligned the assembled transcripts to (1) known homologous proteins (Swiss-Prot), (2) highly conserved sets of eukaryotic genes (CEGMA, BUSCO), (3) available *Platynereis* sequences at NCBI, and (4) a well-curated set of *Platynereis wnt* genes.

**Fig. 3:**
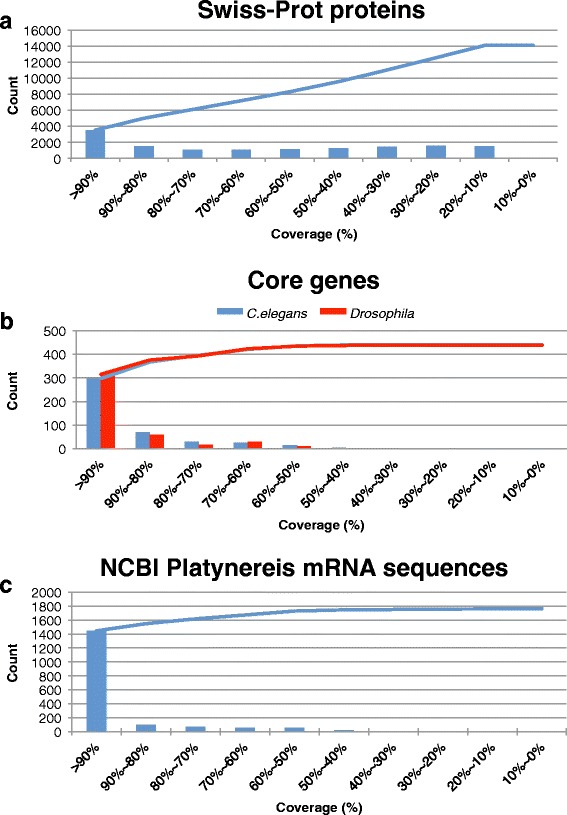
Quality evaluation of the *de novo Platynereis* transcriptome assembly. To evaluate the transcriptome assembly without a reference genome, the assembled transcripts were aligned against curated genes. Coverage refers to the proportion of the curated genes covered by our assembled transcripts. The lines are the cumulative number of genes covered, ordered by the degree of coverage. **a** The *Platynereis* transcriptome aligned to the Swiss-Prot database. 3507 Swiss-Prot proteins were aligned with >90 % coverage and 8335 proteins have at least 50 % coverage. **b** The *Platynereis* transcriptome aligned to the core eukaryotic genes from *Caenorhabditis elegans* and *Drosophila melanogaster*. For both species, ~300 core genes have >90 % coverage. **c** The *Platynereis* transcriptome aligned to cDNA and EST *Platynereis* sequences from NCBI. 1447 of 1775 of the *Platynereis* genes can be assembled with at least 90 % coverage

Alignment of our transcripts against the non-redundant Swiss-Prot database [34] using BLASTX and a cutoff E-value of < 10^−10^ found 8335 transcripts that have at least 50 % coverage (Fig. 3a). Protein sequences from *Caenorhabditis elegans* and *Drosophila melanogaster* retrieved from the CEGMA database [41] were used to test whether a distinct set of core eukaryotic genes can be fully recovered from our assembled transcripts (Fig. 3b). This set of core eukaryotic genes are highly conserved and present in almost every eukaryotic species, and thus serve as a measure of quality for our assembly [42–44]. Most of the core eukaryotic genes in *Platynereis* were assembled to be full-length or nearly full-length when compared to *Caenorhabditis elegans* and *Drosophila melanogaster*. For *Caenorhabditis elegans* core genes, *Platynereis* has 297 fully assembled transcripts (those assembled > 90 % coverage). 421 out of the 458 core genes in *Platynereis* can be assembled to at least 60 % coverage. Similar results were obtained for the core genes of *Drosophila melanogaster* (Fig. 3b). Furthermore, BUSCO analyses were also performed [45] using sets of highly conserved eukaryotic (*n* = 429), metazoan (*n* = 843), and arthropod (*n* = 2675) single-copy orthologs, and found complete assembly for 85, 88, and 73 % of orthologous *Platynereis* genes, respectively (see Additional file 1: Figure S1).

At the time of our analysis, there were 1775 *Platynereis* mRNA sequences submitted to the NCBI nucleotide database, including full-length cDNA and fragmented EST sequences. Aligning our assembly to these sequences finds 1763 out of 1775 sequences have at least one hit using BLASTN with an E-value cutoff of < 10^−10^. Most of these sequences (1447) can be almost completely reconstructed (90 % coverage), indicating the consistency between our assembly and the known *Platynereis* mRNA sequences (Fig. 3c).

As the *wnt* signaling pathway has important roles in early cell specification and segment formation in *Platynereis*, most *wnt* sequences in this species have been manually curated [11, 46]. We chose to use these manually curated sequences to further evaluate the quality of our assembled transcriptome (Table 1). Out of 12 *wnt* genes, we successfully reconstructed *wnt-4, −5, −8, −11, −16* and *-A* with at least 80 % coverage. *wnt-2, −6, −7, −9*, and −*10* were only partially assembled due to the low expression level during early developmental stages.

**Table 1:** The BLASTN alignment of curated *wnt* genes

Wnt genes	FPKM^a^	Length (bp)	% Identity	Aligned Length (bp)	% Coverage
pdum_wnt1	0	1473	0	0	0.0
pdum_wnt2	0.1 (6 h)	1366	98.02	757	55.4
pdum_wnt4	9.0 (10 h)	2503	97.91	2344	93.6
pdum_wnt5_A^b^	15.7 (12 h)	2911	98.25	2906	99.8
pdum_wnt5_B^b^	3.8 (14 h)	2190	97.8	1317	60.1
pdum_wnt6	0.1 (8 h)	1475	94.46	992	67.3
pdum_wnt7	0.2 (6 h)	2312	97.1	1309	56.6
pdum_wnt8	3.5 (10 h)	2139	99.58	1908	89.2
pdum_wnt9	0.2 (10 h)	1169	98.95	382	32.7
pdum_wnt10	0.1 (14 h)	1523	95.1	469	30.8
pdum_wnt11	2.3 (14 h)	1835	97.71	1837	100.0
pdum_wnt16	0.2 (10 h)	1903	96.69	1512	79.5
pdum_wntA	12.7 (8 h)	1840	98.42	1832	99.6

^a^stage with highest FPKM shown

^b^wnt 5a and wnt 5b are different splice variants of the same gene.

We evaluate the quality of our assembly by examining curated *wnt* sequences in *Platynereis dumerilii*. We used BLASTN to compare the similarity between these sequences with our assembly. The coverage refers to the proportion of the curated genes covered by our assembly. Out of 12 *wnt* genes, we successfully reconstructed *wnt4*, *wnt5*, *wnt8*, *wnt11*, *wnt16* and *wntA* with at least 75 % coverage. *wnt2*, *wnt6*, *wnt7*, *wnt9*, and *wnt10* were partially assembled due to the low expression level (<1 FPKM) during early developmental stages

In summary, given that our *de novo Platynerei*s transcriptome was generated exclusively from early embryonic sources it contains a remarkably high fraction of complete gene models. It recovers most core eukaryotic genes (Fig. 3b and Additional file 1: Figure S1), and a major portion of previously submitted *Platynereis* genes that were mostly isolated from later larval stages (Fig. 3c). Even several *wnt* genes that have very low expression levels (Table 1), below the sensitivity of detection by *in situ* hybridization in early stages [11], can be partially reconstructed. Therefore, we conclude that the *de novo Platynereis* transcriptome is of high quality, and may represent every gene that is transcribed at early embryonic stages.

### Functional annotation

**Fig. 4:**
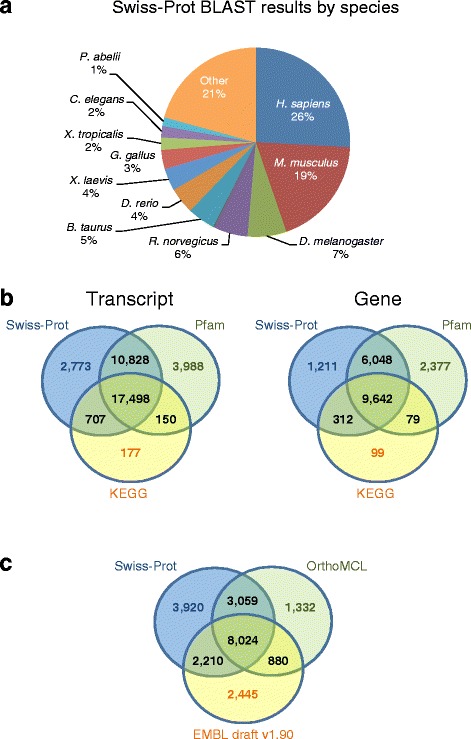
The annotation of the early *Platynereis* transcriptome. **a** *Platynereis* gene models with predicted open reading frames (ORFs) were aligned to the Swiss-Prot databases. >70 % of the *Platynereis* predicted ORFs aligned to vertebrate species. The top five species that the *Platynereis* predicted ORFs aligned to are *Homo sapiens* (26 %), *Mus musculus* (19 %), *Drosophila melanogaster* (7 %) *Rattus norvegicus* (6 %), and *Bos taurus* (5 %). **b** The *Platynereis* transcripts were further functionally annotated using Pfam and Swiss-Prot, to describe potential protein domains, and KEGG to identify pathways, and 9642 genes and 17,498 transcripts are shared by all three categories. 28,326 transcripts and 15,690 genes are associated with both Swiss-Prot and Pfam databases, which are 55 and 56 % of the transcript and gene models with predicted ORFs, respectively. **c** Orthologous analysis was performed using OrthoMCL and the transcriptome data from 18 species including our early *Platynereis* models was collected. Our *Platynereis* models were compared with the EMBL *Platynereis* transcriptome draft version 1.90 which is based on later stages of RNA-seq data. 8024 gene models are supported by all three analyses. (see Results and Methods for details)

To annotate the gene models, we first identified sequences with potential ORFs in the assembly. We found 28,580 genes (51,260 transcripts) that have predicted ORFs > 100 amino acids. The N50 size of these potential coding sequences is 3230 bp. When the gene models with potential ORFs are aligned to the non-redundant Swiss-Prot database including the most rigorously annotated species, 17,213 genes (31,806 transcripts) have at least one hit using E-value cutoff of 10^−10^. Interestingly, over 70 % of the predicted ORFs in *Platynereis* aligned best with vertebrate proteins, including 26 % to human proteins and 19 % to mouse proteins (Fig. 4a), rather than to the evolutionarily more closely related species *Drosophila melanogaster* and *Caenorhabditis elegans* (see phylogeny in Fig. 1b), with only 7 and 2 % of *Platynereis* ORFs aligned, respectively. This is consistent with previous studies and is thought to be due to a dramatic increase of genomic changes including substitutions rates, deletions, and insertions, within the fly and nematode lineages, and less genomic changes within the vertebrate and annelid lineages during evolution [12, 47].

Next, we used databases to identify potential protein domains (Pfam and Swiss-Prot) encoded by *Platynereis* transcripts, and pathways (Kyoto Encyclopedia of Genes and Genomes, KEGG) that these gene models may be involved in to better understand the genes and transcripts with potential ORFs. A total of 431,701 Pfam domains were identified in 18,146 genes (32,464 transcripts) (Fig. 4b). Our previous Swiss-Prot BLASTP search identified 17,213 genes (31,806 transcripts), with 15,690 of these genes (28,326 transcripts) overlapping with Pfam identified genes and transcripts (Fig. 4b). Furthermore, we found that 10,132 genes (18,532 transcripts) are associated with known KEGG pathways and processes. Thus, almost 50 % of all automatically annotated genes (9642) and transcripts (17,498) were found with all three databases, and 2377 (3988), 1211 (2773), and 99 (177) genes (transcripts) were uniquely identified in Pfam, Swiss-Prot, and KEGG, respectively. In summary, automated methods using Swiss-Prot, Pfam, and KEGG databases enabled us to annotate 69.2 % of all genes, and 70.5 % of all transcripts that contain a predicted ORF larger than 100 amino acids.

To further evaluate how many of the 28,580 *Platynereis* gene models with ORFs might be legitimate gene models, we investigated and compared supporting evidence using the previous Swiss-Prot analysis and two additional comparative analyses, finding evidence for 21,870 genes (Fig. 4c). Compared to the Swiss-Prot analysis that found support for 17,213 genes, an OrthoMCL analysis to identify orthologous genes among 18 selected species (see Methods for details) found support for 13,295 genes. Comparison to a *Platynereis* transcriptome assembly recently published by Achim and colleagues [33] found support for 13,559 genes. This is only 62 % of the 21,870 *Platynereis* genes with supporting evidence (47.4 % of gene models with ORFs), suggesting that our focused effort on early stages has significantly increased the number of supported gene models in *Platynereis*. 6710 gene models of the 28,580 gene models with ORFs in our assembly do not have supporting evidence and may constitute either novel *Platynereis* genes or assembly artifacts. However, 4592 genes among these 6710 are unique paralogous genes within *Platynereis,* and may therefore represent novel *Platynereis* gene families.

By comparative analysis we asked how many of the 21,870 supported gene models are conserved in other species from different metazoan taxa of various evolutionary distance (Additional file 2: Figure S2; see Fig. 1b). For genes to be considered conserved, the genes had to satisfy two main criteria: 1) shared genes must represent the best reciprocal blast hits between each species, and 2) the ORFs must share 50 % identity at the protein level. By these stringent criteria the closely related annelids *Capitella telata* and the leech *Helobdella robusta* shared 10,351 genes and 6931 genes with *Platynereis*, respectively. The lower number in leech is likely the result of a derived leech genome [47]. Interestingly, *Platynereis* shared 8900 genes with *Lottia gigantea*, and 8249 with *Crassostrea gigas*, two mollusks more distantly related to *Platynereis* than the leech, suggesting slower genome evolution in both mollusks. Comparing our *Platynereis* gene models with ecdysozoans and deuterostomes we found higher shared numbers with invertebrate deuterostomes such as the echinoderm *Strongylocentrotus purpuratus* (8276), the hemichordate *Saccoglossus kowalevskii* (8482), and the cephalochordate *Branchiostoma lanceolatum* (8144), and lower shared numbers with the ecdysozoans *Daphnia pulex* (6434) and *Drosophila melanogaster* (5669), suggesting much slower genomic changes within the invertebrate deuterostome lineages than in the ecdysozoan lineages consistent with previous studies [12, 47]. This comparative analysis also found 7240 genes, and 7322 genes shared with the vertebrates *Homo sapiens*, and the teleost fish *Danio rerio*, respectively. The lower number of shared genes is likely due to faster genome evolution in the deuterostome lineage leading to the vertebrates, and also to the many additional paralogous genes in vertebrates [48] that obscure/interfere with the best reciprocal blast hit analysis. Comparison with the cnidarian *Nematostella vectensis* found 7717 genes shared with *Platynereis* indicating the deep evolutionary conservation of many protein-coding genes within metazoans [49]. Due to the stringency of this analysis, the number of shared genes between the species represented here is strongly underestimated. This type of systematic analysis with fixed criteria defines the conserved ancestral gene sets present within the early *Platynereis* transcriptome. Although these comparisons point to species that are less and more derived within annelid, spiralian, protostome, and deuterostome model systems, they also suggest that *Platynereis* is one metazoan species that contains a more ancestral gene set.

**Fig. 5:**
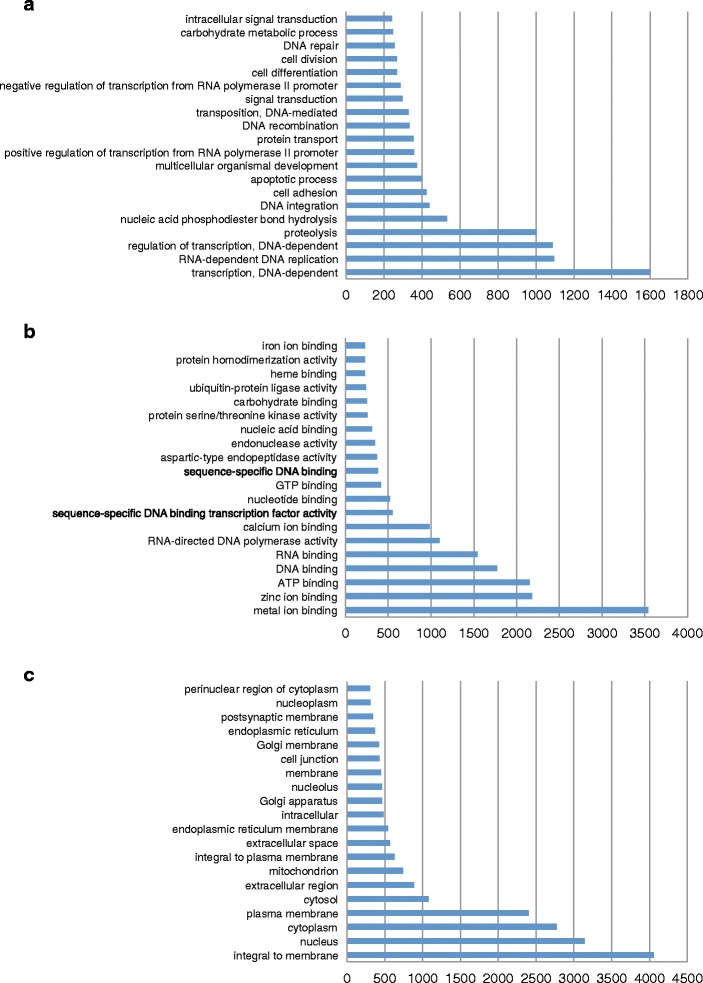
Identification of early *Platynereis* genes in functional subcategories. Gene Ontology (GO) term analysis of the *Platynereis* transcriptome in three categories: **a** Biological process, **b** Cellular component, and **c** Molecular function. The number of genes is indicated for the top 20 GO terms for each category. All individual genes including expression profiles and annotations for each of the 60 categories are listed in Additional file 3: Table S1, Additional file 4: Table S2, Additional file 5: Table S3

We also investigated the gene ontology (GO) terms of homologous genes [50] to begin analyzing the general composition and possible functions of genes in the early *Platynereis* transcriptome. Our transcriptome has 16,498 genes (30,287 transcripts) that are associated with at least one annotated GO term. To find classes of genes that are enriched in our dataset we identified the top 20 GO terms in each of three main categories: biological process, cellular component, and molecular function (Fig. 5; lists of all genes including individual expression profiles are shown in Additional file 3: Table S1, Additional file 4: Table S2, Additional file 5: Table S3). The results demonstrate high enrichment in processes associated with early embryogenesis, including regulation of ‘*gene transcription’* (> 1500 genes), ‘*proteolysis’* (> 1000 genes), ‘*cell adhesion’* (> 400 genes), ‘*signal transduction’* (> 300 genes), and ‘*cell division’* (> 300 genes) (Fig. 5a, Additional file 3: Table S1), cellular components like ‘*golgi*’, ‘*nucleolus*’, and ‘*cell junction’* (Fig. 5b, Additional file 4: Table S2), and molecular functions like ‘*DNA binding’* (> 1800 genes), ‘*serine/threonine kinases’* (> 250), and genes with *‘ubiquitin protein ligase activity’* (> 200 genes) (Fig. 5c, Additional file 5: Table S3). Thus, the GO term annotations enabled us to group and associate our gene models with distinct purposes, identify scores of genes with functions in key processes during early development, and to set the stage for global analyses of differential gene expression during early spiralian embryogenesis.

### Mapping and expression level estimation

To investigate the dynamics of gene expression in early embryonic stages, the normalized expression abundances were estimated for the samples from 2, 4, 6, 8, 10, 12 and 14 hpf. The total number of raw, filtered and mapped reads for each stage is depicted in Fig. 2. 93 % of reads were kept after trimming the adapter sequences and the low quality sections of the reads. Among the remaining reads, 88 % were successfully mapped to the assembled transcripts. The FPKM (fragments per kilobase per million mapped reads) for each sample was obtained by normalization of total mappable reads and transcript length. We also used a scaling normalization method called TMM (trimmed mean of M values) [51] to determine the TMM-normalized FPKM, and a gene or transcript was considered as “expressed” if its TMM–normalized FPKM was greater than 1. An FPKM > 1 was empirically chosen because the onset of gene expression in a single embryonic cell as determined by *in situ* hybridization corresponded generally to an FPKM higher than 5 (see below and previous studies [11, 29], and therefore an FPKM of 1 seemed to be an inclusionary though arbitrary cutoff. Thus, we consider an FPKM > 1 as a reasonable operational definition for a gene being expressed within our datasets. Within the first 14 h of embryogenesis 20,977 genes (34,944 transcripts) are expressed at least once during one of the seven time points sampled. In the one cell zygote at 2hpf, 11,906 genes (16,521 transcripts) are expressed, and these numbers increase until 10hpf (14,961 genes, 20,363 transcripts), and then remain constant at 12hpf and 14hpf. All expressed genes at each stage are listed in Additional file 6: Table S4.

**Fig. 6:**
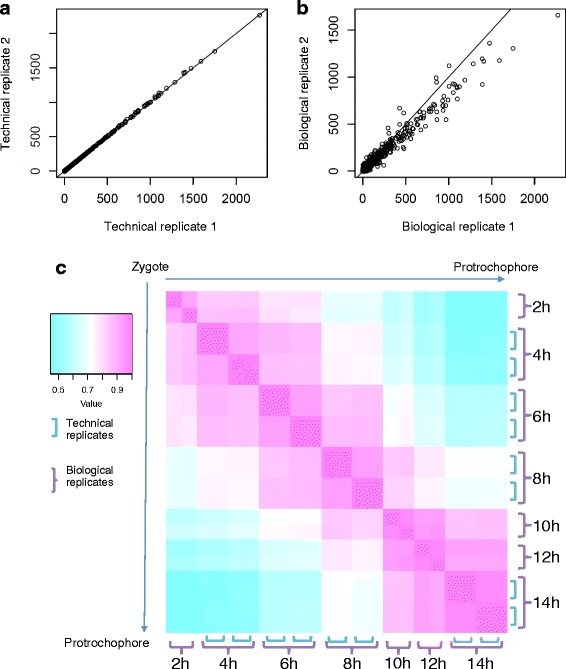
High correlation between replicates suggests tight regulation of gene expression at each stage of *Platynereis* embryogenesis. **a** A Spearman correlation coefficient plot for one set of technical replicates. Each dot represents the expression value (FPKM) in two technical replicates at 6 h post fertilization. The Spearman correlation coefficient is 0.99 between the two technical replicates shown, and is 0.99 or higher for all other technical replicates. **b** A Spearman correlation coefficient plot for biological replicates at 6 h post fertilization. The Spearman correlation coefficient is 0.93 between the two biological replicates shown. The correlation coefficient ranges from 0.91 to 0.97 between all biological replicates. **c** A Spearman correlation coefficient heatmap for all seven stages sampled in our *Platynereis* transcriptome. Biological replicates are denoted with the purple brackets, and the additional technical replicates have blue brackets. The heatmap shows that each individual stage shows the highest correlation to the adjacent stages

In order to understand the reproducibility of gene expression level estimates between samples we calculated the Spearman correlation coefficient between biological and technical replicates for all expressed genes with a predicted ORF. Each of the technical replicates, independent sequencing and calculations of expression levels for the same sample twice, yielded a coefficient of 0.99 showing high reproducibility (example shown in Fig. 6a). Each of the biological replicates, sequencing and calculations of expression levels for two batches of synchronously developing embryos obtained from two different matings but collected at the same time point, yielded an average coefficient of 0.93 (variation between 0.91 and 0.97) showing a remarkably high correlation of global gene expression for any embryonic stage (average shown in Fig. 6b; between all stages shown in Fig. 6c). This is significant as it demonstrates that the expression level for each gene is mostly invariant and tightly regulated for each developmental stage during early embryogenesis.

### Gene expression profiling of early stages in *Platynereis* development

Next, we examined the gene expression profiles of early developmental stages and focused on the expression of genes with predicted ORFs. Among the assembled gene models with predicted ORFs, there are 13,160 genes and 18,940 transcripts that are expressed in at least one of the seven stages. Figure 6c shows the correlation of gene expression between different time points including the correlation between the technical and biological replicates. In general, each individual stage shows the highest correlation with directly adjacent stages. This is significant because it indicates that the selected developmental stages are close enough to share some or most of their expression characteristics with neighboring stages. To determine whether the developmental stages are spaced close enough to capture most transcripts during early embryogenesis, we determined which genes are exclusively expressed in one stage but not in the two neighboring stages. If our sampled time points are close enough, the frequency of highly and exclusively expressed transcripts in any stage should be low, as genes with high levels of expression should be detected in neighboring stages as well. Therefore, the distribution of the highest exclusively expressed transcripts may serve as an indicator of whether the sampling chosen for the transcriptome analysis was insufficient. Indeed, the 30 highest exclusively expressed genes have low levels of expression, range from 1 to 10 FPKM (Additional file 7: Table S5), and are slightly higher at the first and last stages included in the analysis, 2hpf and 14hpf. This might indicate that some genes would continue to be expressed at the next adjacent stages 0hpf and 16hpf. Thus, these low FPKMs of the highest exclusively expressed transcripts in each stage indicate that transcriptional profiling of more intermediate stages eg 7hpf and 9hpf would not capture significantly more novel transcripts. Together, the high correlations between adjacent stages, and the low FPKM of exclusively expressed genes in each stage suggest that our dataset captures important transcriptional dynamics during this early developmental time window.

To gain insights into the stage-specific transcriptional landscape during early embryogenesis we examined the enrichment of KEGG pathways and GO terms for each stage (Additional file 8: Figure S3, Additional file 9: Figure S4; see Methods and Additional files for details). The pathways highly represented include metabolic pathways and cellular functions that generate and shape the transcriptional and translational landscape in embryos, such as the spliceosome, RNA degradation, protein synthesis, and protein degradation. Other enriched functions are associated with the fast embryonic cell divisions during early development, thereby providing insights into the transcriptional regulation of the cell cycle and DNA replication during spiral cleavages. Interestingly, several signaling pathways including Wnt, Hippo, TGF-beta, and Notch/Delta are present in this high frequency group, pointing to potentially important roles for non-cell autonomous mechanisms through these pathways during spiralian embryogenesis. High transcriptional input for key cellular functions implicated in asymmetric cell division, cell polarity and cell adhesion is also detected. Most importantly, enrichments of processes that constitute the early gene regulatory networks including basic and specific transcription factors and the signal transduction machinery potentially involved in cell lineage and cell fate specification in spiral cleaving embryos are found (see also Additional file 9: Figure S4A). Each of these processes or pathways warrants a more in-depth description of specific components and these will be targeted in follow-up studies.

Overall, the enrichment of pathways and processes appears to be similar between different stages with the largest observed differences between the zygote (2hpf) and the other 6 stages. This was also indicated in a correlation analysis (Additional file 8: Figure S3D, Additional file 9: Figure S4D). These differences may be attributed to fast drops in the level of maternally provided transcripts between 2hpf and 4hpf by decay of distinct maternal mRNA species, and to early mRNA synthesis of others. A second, less prominent cluster of processes are mostly enriched in stages after 10hpf and may be involved in the early onset of cell differentiation in some cells, such as the cells that will form the first differentiated cell types involved in motility and light perception [22–24] (Additional file 8: Figure S3B, C, Additional file 9: Figure S4B, C).

With the assembled *Platynereis* early transcriptome, it is possible to dissect the dynamics of the transcriptional landscape between different embryonic stages. We assessed this by (1) determining the number of differentially expressed genes between stages, (2) identifying every gene whose expression is up-regulated or down-regulated between adjacent stages, and (3) defining clusters of genes whose transcriptional developmental profile during the first 14 h of development was similar. Each of these analyses offers unique opportunities to identify overall trends during early spiralian embryogenesis and to detect scores of individual candidate genes whose temporal expression appears to be tightly regulated during development.

**Fig. 7:**
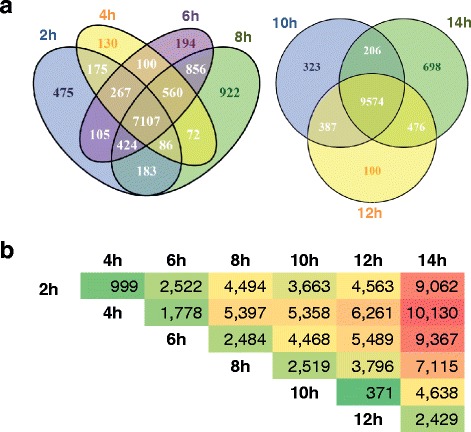
Shared gene expression between stages. A gene is considered expressed if the FPKM > 1. **a** The similarity of expressed genes between two groups, 2–8 h post fertilization (2-8 h) and 10 to 14 h are shown. 7107 genes are shared by all four stages between 2 and 8 h, while 9574 genes are shared by the later three stages. **b** Differentially expressed genes between stages. A gene is considered differentially expressed if the FDR < 0.001. The most differentially expressed genes were found between 8 and 10 h, with 2519 differentially expressed genes. We used a “green-yellow-red” color scheme to represent low, median, and high numbers of differentially expressed genes, respectively (see Results for more details). Lists for individual genes that are significantly down-regulated and up-regulated between each adjacent stage, including expression profiles and annotations, are listed in Additional file 10: Tables S6, Additional file 11: Tables S7, respectively

Systematic analyses of differential gene expression between stages revealed that the first four stages sampled, 2hpf to 8hpf, share the expression of 7107 genes, while 9574 genes are shared by the last three stages, 10hpf to 14hpf (Fig. 7a; Additional file 6: TableS 4; data not shown). This observation may point to a major transition of embryonic gene expression between 8 and 10hpf. Differential gene expression analysis between adjacent embryonic stages found the highest number of differentially expressed genes between 6 and 8hpf, 8 and 10hpf, and 12 and 14hpf (Fig. 7b). However, this method does not make distinctions at the level of expression differences between adjacent stages, and therefore simply shows there is a dramatically changing transcriptional landscape involving thousands of genes from the zygote to the ~330-cell stage.

The RNA-sequencing approach used here can provide a developmental profile for individual genes that show both the transcript level and the timing of the expression. Comparing all expression profiles can reveal individual and global embryonic regulatory mechanisms. This type of analysis begins by identifying all significantly up-regulated and down-regulated transcripts between adjacent stages, including only those genes that increase to a level that is above 10 FPKM in the later stage or decrease from a level that is above 10 FPKM in the earlier stage, respectively. Using these criteria, hundreds of genes have been identified with significant gene expression differences between each adjacent stage (Additional file 10: Tables S6, Additional file 11: Tables S7). While the identity of specific transcripts is useful, this analysis is also informative for discovering larger trends during early spiralian embryogenesis.

During early stages, there is a dominant trend of transcript down-regulation, with 858, 618, 663, and 1041 down-regulated genes between 2–4hpf, 4–6hpf, 6–8hpf, 8–10hpf, respectively. Only 152 and 478 are down regulated between 10–12hpf and 12–14hpf, respectively (Additional file 10: Table S6). Examples for down-regulated genes include genes with previously known early embryonic functions such as Regulators of G-protein signaling (RGS) proteins that are implicated in regulating asymmetric cell division, regulators of the cell cycle like Cyclins and CDK20, and replacing ‘inhibitory’ histone marks necessary for zygotic genome activation. Interestingly, there is a dramatic drop in transcript level for ribosomal proteins between 8 and 10hpf that seems to coincide with a major onset of zygotic gene expression in the embryo.

In an opposing trend, up-regulation of genes is lowest between the first stages (125 genes between 2 and 4hpf), and highest between the last stages (1131 genes between 12 and 14hpf) (Additional file 11: Table S7). Interestingly, the list of genes in the later stages (10hpf to 12hpf) exhibit the onset of gene expression for differentiation genes such as those involved in ciliogenesis, like tektins and axonemal components, as well as hatching enzymes. Between 12 and 14hpf new waves of synthesis of ribosomal and cytoskeleton components may indicate replenishing and remodeling of the machinery for protein synthesis and the cytoskeleton.

**Fig. 8:**
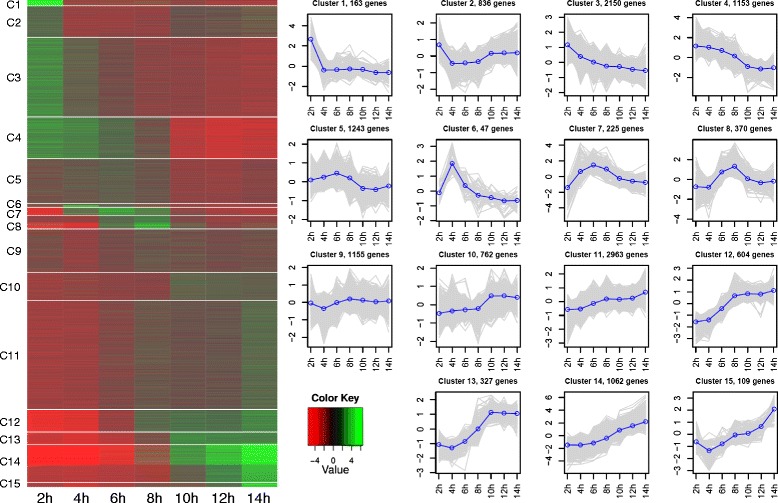
Transcriptional landscape of early *Platynereis* embryogenesis. 13,160 expressed genes (FPKM > 1 in at least one stage) were clustered into 15 groups according to the time series patterns. The developmental expression profile for each individual cluster is shown on the right and includes the number of genes within this cluster. The heatmap on the left is divided horizontally with white lines into the 15 clusters, denoted by C1-C15. Clusters 1–4 correspond to genes with high maternal contribution and Clusters 10–15 are those with later zygotic expression. Clusters 6–8 represent early peaks of zygotic transcription and Clusters 5 and 9 have more stable expression throughout all stages. All individual genes, including expression profiles and annotations for each of the 15 clusters, are listed in Additional file 12: Table S8

To better understand and visualize the global landscape of transcription during the first 14 h of *Platynereis* embryogenesis, a cluster analysis was performed based on similarity of the developmental expression profiles between all 13,160 genes. The clustering analysis grouped each expressed gene within one of fifteen clusters, and detected major general transcriptional transitions during this timeframe (Fig. 8). There are 4302 distinct, maternally provided genes highly or moderately expressed in the zygote at 2hpf (Clusters 1–4), and then the genes decrease in expression level in subsequent stages quickly (Clusters 1 and 2) or slowly (Clusters 3 and 4). Cluster 3 includes the majority of maternally provided genes. Early waves of zygotic transcription are seen in Clusters 6–8, with peaks of expression at 4hpf, 6hpf, and 8hpf. Clusters 5 and 9 are less dynamic, showing more stable expression throughout all stages. Clusters 10–15 (5827 genes) correspond to the zygotically expressed genes, each cluster differing in the activation time point of zygotic transcription. Cluster 11 contains the majority of the zygotically expressed genes. Complete lists of genes within each cluster can be found in Additional file 12: Tables S8.

**Fig. 9:**
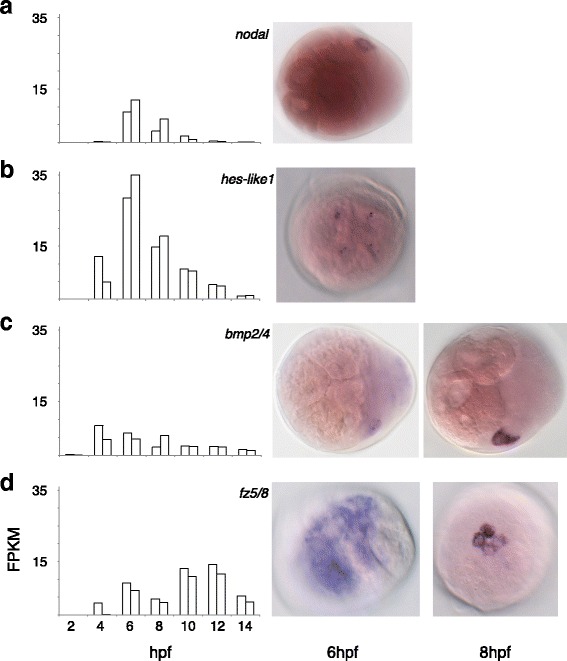
Expression analysis of select developmental regulators in early *Platynereis* development. To confirm the validity of the expression profiles of specific genes from the transcriptome assembly, whole-mount *in situ* hybridization (WMISH) was performed (images on the right). The graphs on the left depict the temporal expression profiles of *nodal, hes-like 1, bmp2/4, and fz5/8*. The expression level is in FPKM, and the two bars at each stage represent biological replicates. **a** Expression of the secreted ligand *nodal*. RNA-sequencing indicates expression is initiated at 6 h post fertilization (hpf). WMISH of 6hpf embryos confirms expression of *nodal* in a single cell at this developmental stage. **b** Expression of the novel Hes family transcription factor *hes-like 1. hes-like 1* is initially expressed at 4hpf with the peak of expression at 6hpf. *hes-like 1* expression can be seen with WMISH on 6hpf embryos in the four animal-most cells. **c** Expression of the secreted ligand *bmp2/4.* RNA-sequencing indicates expression begins at 4hpf and declines slowly over the next ten hours. WMISH confirms expression in early development with expression in one cell at 6hpf and two cells at 8hpf (only one can be seen in the image shown). **d** Expression of the Wnt receptor *fz5/8.* Both biological replicates have expression at 6hpf according to the RNA-sequencing data. With WMISH, expression of *fz5/8* can be seen at 6hpf in the four animal-most cells. Expression of *fz5/8* continues in the animal-most cells at 8hpf, with expression in four cells called the rosette cells

To demonstrate the quality, reproducibility, sensitivity, and utility of the presented RNA-sequencing dataset, we cloned and performed *in situ* hybridization on four important developmental regulators that show early zygotic expression (Fig. 9). *nodal* and *bmp2/4* are both signaling ligands involved in TGF-beta pathways, *hes-1 like* is a transcription factor, and *fz5/8* is a Wnt receptor (previously described in [29]). Comparing biological replicates at each stage demonstrates the reproducibility of our RNA-sequencing dataset as we can measure transcript levels reliably for genes with an expression level above 5 FPKM. This reveals the tight, invariant regulation of the expression level of these genes during embryogenesis. To validate the expression profiles we performed whole mount *in situ* hybridizations in corresponding developmental stages. Intriguingly, *nodal* and *bmp2/4* were found to be expressed in single cells at 6hpf, and also at 8hpf for *bmp2/4* (Fig. 9a, c). Both, *hes-like 1* and *fz5/8* were expressed in the same four cells at the animal pole of the embryo at 6hpf, the former confined to two chromosomal loci within each of the four nuclei (Fig. 9b), and the later with broader expression within the four large animal-pole cells. Each of the four large animal-pole cells divides asymmetrically after 6hpf and forms four small cells at the animal pole at 8hpf. Each of these four smaller cells continues to express *fz5/8* at 8hpf (Fig. 9d). Thus, the presented RNA-sequencing approach is sensitive enough to capture the onset of expression of genes within a single cell or within a few small cells. Furthermore, these results indicate that our stage-specific datasets are sufficient in depth to identify and outline the early gene regulatory networks for a spiral cleaving embryo from zygote to the ~ 330 cell stage.

## Discussion

In this study, we generated the first transcriptomic map of *Platynereis dumerilii* from more than one and a half billion reads sequenced during seven early developmental stages. Such deep sequencing provides an opportunity to explore gene expression profiles with extremely high resolution. As the reference genome is not available yet, a *de novo* transcriptome assembly strategy was employed. We evaluated and demonstrated the high quality of our assembly in terms of the number of full-length reconstruction for the known *Platynereis dumerilii* cDNA sequences as well as homologous genes. The correlation analysis also indicates a high level of reproducibility in our replicates. Our data provides a detailed blueprint of spiralian embryogenesis that will impact future research endeavors in many realms of biology including: (1) fundamental processes of development such as the maternal to zygotic transition and embryonic gene regulatory networks, (2) core cellular processes such as the regulation of cell cycle and asymmetric cell division, (3) comparative analyses of evolution and development such as ancestral reconstruction of maternal contributions to eggs, germ layer specification, and the deciphering of gene regulatory networks that specify lineages and cell type, as well as (4) genome evolution illuminating the content of the ancestral bilaterian transcriptome and genome.

### The early transcriptome: expanding the *Platynereis dumerilii* toolbox

The presented early transcriptome for *Platynereis dumerilii* is an additional valuable molecular resource, adding to several recent efforts to define the broader transcriptome in *Platynereis* [21]. Previously published *Platynereis* transcriptomes were more focused, such as the immune-related transcriptome [31], the neuropeptide complement throughout the life cycle [14], and developmental processes and cell type specification in later larval stages [33, 52]. Our transcriptome analysis is the first to center on early embryogenesis and is therefore the first to focus on spiral cleaving stages, the maternal to zygotic transition, early cell lineage and germ layer specification, and the emergence of the first differentiated cell types. Although the depths and scope of the presented early transcriptome are unprecedented so far, the previous works with different sequencing technologies and a focus on later stages are advantageous to synergistically define the broader *Platynereis* transcriptome. Together, the transcriptomes add to the growing molecular toolbox for this lophotrochozoan spiralian model system by allowing a systems level analysis of any biological process.

### Other spiralian transcriptome resources

In recent years several spiralian/lophotrochozoan transcriptome and genome resources were established. These include the first reported spiralian/lophotrochozoan genomes and/or transcriptomes for two plathyhelminths *Schistosoma* species (*Schistosoma mansoni* [53] and *Schistosoma japonicum* [54]), the annelids *Capitella teleta* and *Helobdella robusta* [47], the mollusks *Lottia gigantea* [47], *Aplysia californica* [55], *Ilyanassa obsoleta* [56], *Crepidula fornicate* [57], the polyclad flatworm *Maritigrella crozierie* [58], and the annelids *Pristina leidyi* and *Hermodice carunuculata* [59, 60], with most sequencing derived from mixed stage cDNA-libraries. In addition, several recent extensive phylogenomic studies contributed transcriptomes from dozens of annelid species [4, 19, 61, 62]. Although not focused on early stages, these transcriptomes provide an enormous resource to investigate the conservation and evolution of protein coding genes within annelid phylogeny. Stage-specific transcriptome sequencing has been done for three selected stages (1 cell, 2 cell and 32 cells) for the pond snail *Lymnaea stagnalis* [63], and stages throughout the life cycle of the pacific oyster *Crassostrea gigas* [64] including a similar set of early embryonic stages to the presented *Platynereis* dataset. Although the oyster sequencing data is not as comprehensive as our *Platynereis* data, it will be an exciting source to unravel conserved and derived molecular mechanisms between the spiral cleaving programs of mollusks and annelids.

### Identifying a comprehensive “Parts” list for early spiralian embryogenesis

The depth and scope of the presented sequencing effort was designed to capture every potentially expressed transcript during the first 14 h of *Platynereis* development. The correlation analysis between adjacent stages (Fig. 6c) and the low frequency of exclusively expressed, stage-specific genes (Additional file 7: Table S5) suggest that only a few transcripts below 2–3 FPKM might have been missed in this dataset. Thus, the > 20,000 transcribed genes with an expression level of > 1FPKM (Additional file 6: Table S4) likely represents a comprehensive early transcriptome, yielding an extensive parts list for every process that comprises spiralian embryogenesis. To subdivide the extensive parts list, 60 GO term classes were used to classify each gene further and provide new parts lists for each process or function within the annotated transcriptome. Thus, these identified and categorized genes comprise a blueprint for early *Platynereis* development, will be instrumental in dissecting spiralian embryogenesis in *Platynereis*, and will facilitate cross species comparisons among spiralians and other metazoans.

### Dynamics of the early *Platynereis* transcriptomes during spiral cleavages (I to V)

The *Platynereis* transcriptome described here was assembled from deep sequencing to allow for the opportunity to (1) explore gene expression profiles with extremely high resolution, and (2) define broader trends in overall gene transcription. In general, the transcriptional landscape is very dynamic with ~ 200–2500 genes being differentially expressed between each stage. Further, we found that more genes are down-regulated between each adjacent stage during the first four stages sampled (2hpf to 8hpf), while up-regulation of genes between adjacent stages is more prominent between the later stages (10hpf to 14hpf). This suggests a major switch in the transcriptional landscape between 8 and 10hpf, and this timing is corroborated by the cluster analysis of differentially expressed genes (Fig. 8). We believe this marks the transition of maternal to zygotic gene transcription in *Platynereis* embryogenesis, the Maternal to Zygotic Transition (MZT) [65].

The cluster analysis identified 15 different clusters of genes that show differing temporal onsets of transcription and uncovered intriguing dynamics of transcriptional regulation during spiral embryogenesis (Fig. 8). 8hpf has been identified as a truly transitional stage with several maternal genes still expressed (Clusters 4 and 5), and several zygotic genes already expressed (Clusters 11 and 12). After the transition from 8hpf to 10hpf, the maternal gene clusters are nearly gone, and zygotic gene clusters are either more elevated or new expression is observed (Clusters 2, 10, 13, and 14). Interestingly, the core spiral cleavage program is completed by 8hpf, suggesting that the spiral cleavage program itself might be largely under maternal control. However, several unique, early stage-specific peaks (Clusters 6 and 7), and several early onset genes in Clusters 11 and 12 could contribute some zygotic regulation during spiral cleavage stages. In summary, the maternal to zygotic transition in this spiral-cleaving embryo is comprised of several waves of zygotic expression, with the major transition to the zygotic landscape after 8hpf.

I. Maternal contribution to a spiralian egg
  The first stage included in our transcriptome was 2hpf, the *Platynereis* zygote. There are four maternal clusters of genes (Clusters 1–4) that share the trend of moderate to high enrichment in the zygote and then a decrease in level of expression during consecutive stages (Fig. 8). While the early stages, 2hpf to 8hpf, share a high number of expressed genes (7107 genes), the KEGG and GO term enrichment analysis between stages found that the 2hpf zygote differs substantially from the other early stages (Additional file 9: Figure S4, Additional file 10: Tables S6, Additional file 12: Tables S8, Additional file 13: Table S9, Additional file 14: Table S10). For example, in the *Platynereis* zygote there are enrichments for functions associated with nucleotide binding, protein degradation and synthesis, and the cell cycle, and this is consistent with a previous analysis that compared maternal contributions of a mollusk egg, the snail *Lymnaea stagnalis* to different bilaterians [63]. Although considered mostly housekeeping functions, the dynamics of these gene cohorts facilitates new insights into the orchestration of genes in early development and warrants follow-up studies. For example, individual genes in Cluster 1 (136 genes) that show an extreme decrease in expression in the early stages, like the RHOGTP-activating enzymes and several Regulator of G-protein signaling molecules implicated in regulation of early cell division in other early embryos [66–68], will be especially interesting to study (Fig. 8; Additional file 12: Table S8). Future analysis with this transcriptome data for the *Platynereis* zygote can begin to answer fundamental questions such as ‘What is inside the egg?’ or ‘How similar is the egg composition among different metazoans?’ or ‘What are conserved and novel maternally provided components between different egg types?’. This can open research avenues to further define maternal contributions and enable cross species comparisons, promising general insights into early embryogenesis, and evolution and development.
II. Clearance of maternal transcripts in a spiralian embryo
  Clearance of maternal transcripts is one of the two major mechanisms for the transfer of developmental control during the maternal to zygotic transition in embryos [65], and this clearance is visible in the *Platynereis* transcriptome presented here. The four clusters with maternally provided genes (Clusters 1–4, Fig. 8) each show different degrees and timing in the down-regulation of maternal transcripts. In Cluster 1, the maternal transcripts are cleared rapidly between 2 and 4hpf. In contrast, in Cluster 4, the maternal transcripts are cleared later and much more slowly. This suggests the possible existence of several different mechanisms for maternal clearance in *Platynereis*.
  Early clearance mechanisms often target regulators of the cell cycle [65]. In this regard it is interesting that transcripts for a cdc25/string-like gene are dramatically decreased from above 250 FPKM to below 15 FPKM between 2 and 4hpf. Our cluster analysis also suggests a final clearance step for distinct transcript species between 8 and 10hpf (Fig. 8). Interestingly, this includes a very pronounced decay of many ribosomal transcripts (Additional file 10: Table S6) that may constitute a defining signature for the end of maternal clearance in *Platynereis*. Thus, the transcriptional landscape for early *Platynereis* development shown here may be used for the discovery and regulation of clearance mechanisms, and for understanding the shift from maternal to zygotic control of the cell cycle in early embryos.
III. Zygotic activation of gene expression
  The second major mechanism for the transfer of developmental control during the maternal to zygotic transition in embryos constitutes the zygotic activation of gene expression [65]. Similar to several recent studies that utilize RNA-sequencing or microarray approaches to investigate the MZT in various other species, *Platynereis* embryos seem to exhibit early waves of zygotic gene expression: 47 genes show peak expression at 4hpf, 225 genes at 6hpf, and 370 genes at 8hpf (Fig. 8; Additional file 12: Table S8). Concurrent with the later peak, a first larger cohort of genes (Clusters 9, 11 and 12; Fig. 8) exhibits a moderate increase in overall gene expression level between 6 and 8hpf, followed by a more pronounced increase of a second major cohort of genes between 8 and 10hpf (Clusters 13 and 14, Fig. 8). Thus, the major wave of zygotic transcription appears to coincide with the end of the 6^th^ or 7^th^ cycle of cell division after 8hpf, and at the transition to bilaterally symmetric cell divisions in some cell lineages in *Platynereis* [22, 24]. Although more experiments are necessary to identify if these changes in transcript level correspond to active zygotic gene expression [65], the presented developmental transcriptional landscape provides hints for future studies.
IV. Early small waves of zygotic expression
  Our study identified several early small waves of zygotic expression comprising three clusters (Clusters 6, 7, and 8, Fig. 8; Additional file 12: Table S8). The three clusters contain 642 genes including many transcription factors such as members of the glia cell missing (GCM)-, forkhead (Fox)-, paired box (PRD)-, hes-like, and zinc finger-like gene families that will be further described in subsequent studies. These small, early waves of transcription have been found in several species (for review see [65]), and it will be interesting to see whether common molecular mechanisms will be found. However, many of the early expressed genes appear to be more derived genes within their particular conserved gene families. For example, preliminary phylogenetic analyses of early expressed transcription factors identified at least seven derived Fox-like, two derived GCM-like, three derived Hes-like, and three derived PRD-like genes that defy easy classification within their conserved gene families (Additional file 12: Table S8; data not shown). This observation is in agreement with recent findings that novel, lophotrochozoan-specific homeobox genes are preferentially recruited to and utilized in early development in the oyster *Crassostrea gigas* [69]. Paps and colleagues (2015) suggest the observations with *Crassostrea* support an hourglass or phylotypic stage model of developmental evolution, predicting more novelty and evolutionary lability in the regulation of early developmental stages. Rapid divergence and novelty in transcriptional regulation of early embryogenesis is further supported by the many divergent and derived GATA transcription factors that exist in nematodes like *C. elegans*, with many crucial functions in the early endo- and mesodermal gene regulatory networks [70–72]. Consistent with these observations is also a recent comparative study that found that early zygotic genes in zebrafish exhibit little evolutionary conservation and are composed of mostly novel genes [73]. Thus, these observations in *Platynereis* (this study), *Crassostrea* [69], *C. elegans* [71], and zebrafish [73], may lend support to the phylotypic stage model of developmental evolution (see also Levin and colleagues [52] for alternative observations), and may also reflect recent notions that the conserved pattern of spiral cleavage might be regulated differently within and between different spiralian clades [74].
V. Later major waves of zygotic expression
  In *Platynereis*, the major waves of zygotic transcription begin between 6 and 8hpf with a modest increase in expression in ~3500 genes (Clusters 11 and 12, Fig. 8). This major wave of zygotic transcription is shortly followed by another wave of zygotic expression between 8 and 10hpf (Clusters 10, 13, and 14). The second wave of zygotic expression includes ~2100 genes, most of which have a higher increase in expression level compared to the waves between 6 and 8hpf. The gene clusters from the two major waves of zygotic expression include many components of gene regulatory networks including conserved transcription factors and signaling components (see below). Many critical components of major pathways are expressed like receptors and ligands of Wnt pathways such as *frizzled 1/2/7, sFRP1/2/5, wnt-4* and *wnt-5* [11, 29]. These clusters also harbor many genes that support the formation of the first differentiating cell types in the *Platynereis* embryo such as early onset ciliogenesis genes (Additional file 12: Table S8). Thus, these clusters comprise of many crucial developmental regulators and the first differentiating gene cascades, all of which reflect the hybrid nature of the developing protrochophore that is composed of developing cell lineages as well as the first functional cell types [22, 24]. Due to the higher amplitude of the major wave of zygotic transcription between 8 and 10hpf, we suggest that this period represents the major transition to zygotic control in the *Platynereis* embryo.

### Comparison of the maternal to zygotic transition in *Platynereis* to other species

The fine-scale transcriptional landscape of the MZT has been reported recently for several animal model systems (reviewed in [65]). Detailed comparative analyses have just begun [73], but are complicated by the many unique early developmental modes that need to be considered. Different developmental modes greatly change the early transcriptional landscape, especially the time point and scale of zygotic gene activation, which can differ depending on size of the embryo, the speed of early cell divisions, and the time when gastrulation occurs. Early zygotic gene expression has been reported as early as the formation of the male and female pronuclei in the nematode *Ascaris suum* and in the 1-cell mouse embryo (both exhibit very slow development) [75–77], but also in the 1-cell stage of the much faster developing sea urchin embryo [78]. Many animal embryos exhibit early and small waves of zygotic expression followed later by larger zygotic waves of gene expression [73, 79–85] similar to what is seen with our *Platynereis* dataset. The scale of early and late zygotic transcription involving hundreds to several thousands of genes, respectively, appears to be similar between *Platynereis* and other animal model systems [65], and in all animal embryos, including *Platynereis,* the major zygotic activation is well underway before the beginning of gastrulation.

### Systems-level insights into the early embryogenesis of *Platynereis*

Remarkably, the transcript level for individual genes in biological replicates at the same stage was very similar (Figs. 6b, c and 8), highlighting three important aspects of this study: (1) the accuracy of the RNA-sequencing approach to capture the level of thousands of transcripts reliably within a sample, (2) the suitability, amenability and strength of the *Platynereis* model system that enables the collection of thousands of synchronized developmental stages [6] and subsequent sequencing library preparations without necessary amplification steps, and (3) the apparent tight regulation within developing *Platynereis* embryos that determines the exact level of each transcript. The observed level of accuracy seen in the dataset suggests each mechanism in early development is tightly controlled on the transcriptional level.

Thus, this study accurately captures and dissects the changing genome-wide transcriptional landscape between a one-cell zygote to a hatched and rotating 330-cell stage protrochophore that exhibits the first differentiated cell types. Further, the *Platynereis* dataset presented here reveals the complete transcriptional input for any process related to early spiralian embryogenesis, whether considered ‘house keeping’ or ‘developmental regulative’ functions (Fig. 5, Additional file 3: Table S1, Additional file 4: Table S2, Additional file 5: Table S3, Additional file 8: Figure S3, Additional file 9: Figure S4). For example, there is a prominent and coordinated up-regulation of ribosomal transcripts and components of microtubules between 12 and 14hpf, and expectedly, up-regulation of ciliogenesis genes between 8 and 12hpf when the cells that will form a ciliated ring stop dividing and start to differentiate [22, 24, 28]. Furthermore, our dataset identified the evident expression of several key developmental pathways such as Wnt, Hippo, Notch, and TGF-beta during early embryonic stages. These findings will enable future studies to begin systems-level approaches to understand the coordinated regulation of hundreds of components that constitute these individual pathways throughout early development. We demonstrate that by determining the transcriptional profiles between close enough stages one can capture the outline of the underlying gene regulatory networks in embryos comprehensively.

### Defining the gene regulatory networks in early spiralian development

One of the motivations for this study was to identify all components of each gene regulatory network (GRN) that operates during early spiralian embryogenesis in the annelid *Platynereis dumerilii* (Additional file 6: Table S4). The functional annotation of our dataset allowed us to identify hundreds of transcription factors and several signaling pathways that may constitute the embryonic GRNs. Furthermore, our transcriptional developmental profiles for each gene expressed identifies the exact time during development when a gene is turned on and when its transcripts are depleted. The developmental profiles and the analysis to determine each gene up-regulated or down-regulated between adjacent stages helped to pinpoint the onset of expression for many transcription factors, signaling ligands, receptors, and intracellular components of signaling pathways during early spiralian development (Additional file 10: Tables S6, Additional file 11: Tables S7, Additional file 12: Tables S8).

To validate the data from the RNA-sequencing approach, specific genes that are important developmental regulators were cloned and their spatial and temporal expression was determined in early *Platynereis* embryos (Fig. 9). The showcased genes have low to moderate levels of expression, with peak values between 9 and 30 FPKM, to highlight the ability and sensitivity of the data to capture lowly expressed transcripts. Each biological replicate has similar FPKM values, and we believe that any observed differences are due to slightly accelerated development in some batches of our embryos caused by minute local fluctuations in our temperature-controlled culture room. Using whole mount *in situ* hybridization to localize the transcripts, the onset of expression can be seen in a single cell (*nodal*, *bmp2/4*; Fig. 9a, c), or a few cells (*hes-like 1*, *fz5/8*; Fig. 9b, d). The expression can even be visualized at the two chromosomal loci in individual nuclei (*hes-like 1*, *bmp2/4*; Fig. 9b, c). These results demonstrate the transcriptional landscape of early *Platynereis* development produced by RNA-sequencing is reproducible, sensitive and predictive for low to moderate level expression of developmental regulators, and therefore can provide a comprehensive map outlining embryonic GRNs in this spiralian embryo.

## Conclusions

In summary, we have provided the first comprehensive transcriptome study of spiralian embryogenesis for the marine annelid *Platynereis dumerilii,* reporting the sequence, annotation, and stage-specific expression level for 28,580 genes. The unprecedented depth in sequencing of seven developmental stages identified the dynamic and invariant transcriptional landscape from the one cell zygote to ~330 cell stage, including the maternal contribution of 11,904 genes, the maternal clearance of over 4000 expressed genes, and both the minor and major waves of zygotic expression. Quantifying the expression levels for each expressed gene at each developmental stage analyzed captures the transcriptional input into every biological process during spiralian embryogenesis and outlines the gene regulatory networks specifying cell lineages and germ layers in the *Platynereis* embryo. Our gene models and functional annotation in this spiralian/lophotrochozoan model system may serve as a valuable resource to further decipher the gene regulatory networks patterning cell lineages and cell fates during early spiralian development, providing crucial systems-level data to infer conserved and novel molecular features of early bilaterian development and evolution.

## Methods

### *Platynereis dumerilii* culture, embryo collection, cDNA preparation, and sequencing

All *Platynereis* embryos were obtained from a single breeding culture maintained at Iowa State University according to standards and protocols established at www.platynereis.de. In order to ensure consistent developmental timing and reproducibility, embryos were kept in an 18 °C incubator where the temperature is continuously monitored. Newly fertilized eggs were monitored for quality including formation of jelly coat and normal cleavage patterns before selection for RNA collection. Biological replicates for each time point were obtained from independently fertilized batches of embryos developing synchronously under identical conditions. Embryos were collected at 2, 4, 6, 8, 10, 12, and 14hpf and homogenized in Trizol (Ambion). In each case ~100–200 embryos were left behind as quality controls to be monitored for normal development for 48 h before proceeding to RNA extraction from Trizol in accordance with the manufacturer’s protocol. Extracted RNA was treated with the RNase-free DNase set (QIAGEN), purified with the RNeasy Mini Kit (QIAGEN), and checked for RNA degradation on a 1 % agarose gel. Additional total RNA quality controls using the Bioanalyzer system (Agilent), preparation of each barcoded Illumina mRNA-seq library, and Illumina deep sequencing with 75 bp–100 bp paired-end reads (4 samples per lane) were performed by the Genome Sequencing & Analysis Core Resource at Duke Institute for Genome Sciences and Policy using an Illumina HiSeq sequencing system.

### Read processing and *de novo* assembly

The raw reads were preprocessed with Trimmomatic [86] to remove the adapter sequences and low quality regions. Bases were removed from both ends if the Phred score was lower than 20. A sliding window of 4 was used to continue removing bases with the threshold of an average score of less than 20. The quality of the preprocessed reads were analyzed and visualized by FASTX-Toolkit [87]. To create unified gene models, all samples were combined together and assembled in a single pass. Trinity’s *in silico* normalization utility was used, with a k-mer size of 25, to reduce the memory requirement and improve the running time. Trinity assembled the normalized reads into 357,961 transcripts with an N50 size of 2331 bp. The digital normalization and assembly were performed on the Stampede high performance computing system, which is a part of Texas Advanced Computing Center (TACC) [88]. With the final assembly, transcripts with more than 95 % identity were further clustered using CD-HIT’s “cd-hit-est” program [89]. From this 273,087 transcripts were obtained with an N50 size of 1466 bp. These transcripts belong to 193,310 genes and 50,237 of the genes are longer than 1000 bp.

### Functional annotation of the *Platynereis* transcriptome

The putative coding regions of the final transcripts were predicted using TransDecoder [90], which can extract the long ORFs and estimate the posterior probability of all six possible open reading frames using the Markov Model. The likely protein sequences were aligned to the Swiss-Prot database [91] using BLASTP search with options “-evalue 1e-10 -max_target_seqs 1”. We also made use of the curated gene ontology and eggNOG information provided by Swiss-Prot FTP site (ftp://ftp.uniprot.org/pub/databases/uniprot). The Pfam [35] domains of the protein sequences were predicted using default parameters in the hmmscan program in HMMER [92]. For the KEGG pathway analysis [93], KEGG’s REST APIs were used to download the KEGG curated protein sequences. A BLASTP search (E-value < 10^−10^) was employed to identify the homologous KEGG genes and the associated pathways. An R package, VennDiagram, was used to count the number of annotated genes and plot the Venn diagram. All annotation information was uploaded to a MySQL database and the data used in enrichment analyses were filtered by custom SQL commands.

To perform whole transcriptome comparison to other metazoan species, potential orthologous groups were identified using the OrthoMCL pipeline [94]. Except for our *Platynereis dumerilii* gene models, protein sequences were collected from 17 species: *Capitella teleta* [95], *Helobdella robusta* [95], *Lottia gigantea* [95], *Crassostrea gigas* [64], *Daphnia pulex* [95], *Tribolium castaneum* [96], *Drosophila melanogaster* [97], *Strongylocentrotus purpuratus* [98], *Saccoglossus kowalevskii* [95], *Branchiostoma floridae* [95], *Danio rerio* [99], *Xenopus tropicalis* [95], *Homo sapiens* [99], *Nematostella vectensis* [95], *Amphimedon queenslandica* [95], *Trichoplax adherens* [95] and *Monosiga brevicollis* [95]. Only the longest splicing isoform for each gene was included in the comparison. An all-against-all BLASTP search was performed using 1e-05 cutoff. Any hits with less than 50 % identity match were removed. The orthologous relationships were confirmed if genes are the reciprocal best hits for any two species. 40,206 groups were identified using the MCL clustering algorithm [100], and 32,482 groups have at least two species.

Our models were also compared with another transcriptome assembly from *Platynereis* preferentially generated by sequencing mixed and later larval stages [33] that includes 102,433 transcripts. BLASTN was used to search our gene models at the nucleotide level against the other assembly with an E-value cutoff 1e-20.

### Assessment of the *Platynereis* transcriptome assembly

To evaluate the quality of our assembly, the assembled transcripts were aligned to known orthologous models from the Swiss-Prot database, the NCBI *Platynereis* database, the CEGMA database, and the BUSCO datasets [45] The completeness was measured as the percentage of target sequences aligned to the *Platynereis* gene models in the transcriptome presented here. BLAST searches and an in-house Perl script was implemented to parse the BLAST results and to calculate the coverage. Two CEGMA datasets (*Caenorhabditis elegans* and *Drosophila melanogaster*) were downloaded from CEGMA website (http://korflab.ucdavis.edu/datasets/cegma/#SCT5). For BUSCO analysis, three BUSCO datasets (Arthropods, Metazoans and Eukaryotes) were downloaded from BUSCO website (http://busco.ezlab.org/). The BUSCO main script (BUSCO_v1.1b.py) was used to generate assessment results with option “-m trans”.

### Mapping and expression level estimation

The read count of each sample was calculated by RSEM, which used Bowtie [101] as an aligner to map the raw reads back to the assembled transcripts. The number of aligned reads was calculated using SAMtools “flagstat” program [102]. An R package “edgeR” [51] was used to incorporate the TMM scaling factor and to calculate the differentially expressed genes. The differentially expressed genes were filtered by False Discovery Rate (FDR) adjusted *p*-value < = 0.001. For the clustering analysis, only genes with an FPKM > 1 in at least one stage were selected and the genes were clustered into 32 groups using median-centered log_2_-transformed FPKM with the R function “hclust” (hierarchical clustering). The clusters were further manually combined into 15 clusters and were ordered by the expression patterns using a custom Perl script. The heatmap was generated by the R function “heatmap.2”. For the correlation analysis, the pairwise Spearman correlation between all biological and technical replicates was calculated using the R function “cor” and the correlation scatter plots were visualized using the R function “plot”.

### Cloning genes and whole-mount *in situ* hybridization

All cloning, anti-sense RNA probe preparation, and whole-mount *in situ* hybridizations (WMISH) were performed as previously published [11, 29]. In short, gene sequences were found using the assembled transcriptome, primers designed with Primer3 [103], stage-specific cDNA was used with the gene-specific primers to amplify the target gene. Standard cloning procedures were used to clone the PCR-amplified gene target into a vector that allows for anti-sense RNA transcription. Ages of embryos used for WMISH was based on the expression profile for the specific target. WMISH procedure was performed as previously described [11, 29].

## Abbreviations

BLAST, Basic Local Alignment Search Tool; bp, base pair; CEGMA, Core Eukaryotic Genes Mapping Approach; EST, Expressed sequence tag; FPKM, Fragments Per Kilobase per Million mapped reads; GO, gene ontology; GRN, gene regulatory networks; hpf, hours post fertilization; KEGG, Kyoto Encyclopedia of Genes and Genomes; MCL, Markov Cluster Algorithm; MZT, maternal to zygotic transition; nt, nucleotide; ORF, open reading frame; TMM, Trimmed Mean of M-values; WMISH, whole-mount *in situ* hybridization

## Additional files

Additional file 1: Figure S1. Assessment of the *Platynereis* transcriptome assembly. To evaluate the completeness of our assembly, our gene models were compared with eukaryotic, metazoan, and arthropod universal single-copy orthologs using BUSCO. For the eukaryotic subset, the majority of eukaryote orthologs, 85 %, were completely assembled (blue), 6 % were partially assembled (crimson), and 8 % could not be identified in our assembly (green). The subsets of metazoan and arthropod orthologs have 88 and 73 % completeness, respectively. (PDF 30 kb) Additional file 2: Figure S2. Conservation of early *Platynereis* gene models in various metazoan species. Our early *Platynereis* transcriptome comprises of 21,870 gene models with ORFs >100 amino acids that are supported by the Swiss-Prot database, the OrthoMCL analysis, or the EMBL draft 1.90. To further understand the gene/protein evolution of these *Platynereis* gene models, we constructed orthologous groups among 18 selected species (see OrthoMCL analysis in Methods for details), and determined the number of shared genes between *Platynereis* and specific groups of species based on two stringent criteria: 1) shared genes must represent the best reciprocal blast hits between each species, and 2) the ORFs must share 50 % identity on the protein level. This figure shows Venn diagrams of the number of genes shared by the three selected species (orange), the number of genes shared between *Platynereis* and one of the species (yellow and crimson), and the number of genes in *Platynereis* only (blue). Each of the seven sets shown here contains *Platynereis* and two different species of similar phylogenetic distance. The 7 sets include: (A) *Platynereis dumerilii*, and two annelids, the leech *Helobdella robusta* and the bristle worm *Capitella teleta*. (B) *Platynereis dumerilii*, and two spiralian mollusks, the limpet *Lottia gigantea* and the pacific oyster *Crassostrea gigas*. (C) *Platynereis dumerilii*, and two ecdysozoans, the crustacean water flea *Daphnia pulex* and the insect fruit fly *Drosophila melanogaster*. (D) *Platynereis dumerilii*, and two invertebrate deuterostomes, the hemichordate *Saccoglossus kowalevskii* and the cephalochordate lancelet *Branchiostoma floridae*. (E) *Platynereis dumerilii*, and two deuterostome ambulacrarians, the acorn worm *Saccoglossus kowalevskii* and the echinoderm sea urchin *Strongylocentrotus purpuratus*. (F) *Platynereis dumerilii*, and two deuterostome vertebrates, the teleost zebrafish *Danio rerio* and the mammal human *Homo sapiens*. (G) *Platynereis dumerilii*, and two metazoans, the cnidarian sea anemone *Nematostella vectensis* and the chordate human *Homo sapiens*. (PDF 102 kb) Additional file 3: Table S1. List of all genes and expression profiles for each of the 20 classes in the Gene Ontology category ‘Biological Process’ (related to Fig. 5). For the top 20 enriched GO terms in the biological process namespace, we list all genes associated with these GO terms (one GO term per sheet). The expression profiles and the annotation information based on the BLAST results against the Swiss-Prot database are also included. (XLSX 999 kb) Additional file 4: Table S2. List of all genes and expression profiles for each of the 20 classes in the Gene Ontology category ‘Cellular Component’ (related to Fig. 5). For the top 20 enriched GO terms in the cellular component namespace, we list all genes associated with these GO terms (one GO term per sheet). The expression profiles and the annotation information based on the BLAST results against the Swiss-Prot database are also included. (XLSX 2059 kb) Additional file 5: Table S3. List of all genes and expression profiles for each of the 20 classes in the Gene Ontology category ‘Molecular Function’ (related to Fig. 5). For the top 20 enriched GO terms in the molecular function namespace, we list all genes associated with these GO terms (one GO term per sheet). The expression profiles and the annotation information based on the BLAST results against the Swiss-Prot database are also included. (XLSX 1675 kb) Additional file 6: Table S4. List of all genes and expression profiles for each stage. A gene is considered expressed if the FPKM is larger than 1. All expressed genes for each stage and their annotation information based on the BLAST results against the Swiss-Prot database are included here. (XLSX 8428 kb) Additional file 7: Table S5. Highest exclusively expressed genes at each stage. The 30 highest exclusively expressed genes at 2, 4, 6, 8, 10, 12, and 14 hpf are shown including their calculated FPKM (see results for more details). (XLSX 15 kb) Additional file 8: Figure S3. KEGG enrichment analysis at each stage. The number of KEGG pathways associated with the top 5000 expressed genes at each stage was counted. Hierarchy clustering was used to classify these pathways and three distinct groups were observed: (A) High frequency (> 2^6^), (B) Medium frequency (2^4^ ~ 2^6^), and (C) Low frequency (< 2^4^). (D) All stages shared similar associated KEGG pathways (correlation coefficient >0.85). Adjacent stages have higher correlation with the exception of 2hpf, which is not similar to any other stage. (PDF 661 kb) Additional file 9: Figure S4. Gene Ontology enrichment analysis at each stage. The top 5000 expressed genes at each stage were selected and their associated GO terms were shown by their namespaces: (A) Biological process, (B) Cellular component, and (C) Molecular function. (D) All stages show similar GO term enrichment patterns (correlation coefficient > 0.8). Similar to the KEGG pathways analysis, the 2hpf GO terms are less similar to the other six stages. (PDF 632 kb) Additional file 10: Table S6. List of down-regulated genes between adjacent stages. Only genes that exhibit an FPKM of 10 or higher in the earlier stage are shown. The list is ordered by FDR (cutoff is 0.001) and includes the expression values and the annotated protein name. (XLSX 143 kb) Additional file 11: Table S7. List of up-regulated genes between adjacent stages. Only genes that exhibit an FPKM of 10 or higher in the later stage are shown. The list is ordered by FDR (cutoff is 0.001) and includes the expression values and the annotated protein name. (XLSX 123 kb) Additional file 12: Table S8. List of genes in each cluster (related to Fig. 8). We classified all expressed genes into 15 clusters and all genes within the clusters are listed here. The expression profile and the annotation information are also included. (XLSX 1448 kb) Additional file 13: Table S9. KEGG enrichment in each cluster (related to Fig. 8 and Additional file 12: Table S8). For each cluster, we list the top 30 enriched KEGG pathways. These KEGG pathways are ordered by the number of pathways associated with the genes in the clusters. (XLSX 17 kb) Additional file 14: Table S10. Gene Ontology term enrichment in each cluster (related to Fig. 8 and Additional file 12: Table S8). For each cluster, we list the top 30 enriched GO terms. These GO terms are ordered by the number of GO terms associated with the genes in the clusters. (XLSX 17 kb)
