# Supplementary figures and images for: A transcriptional blueprint for a spiral-cleaving embryo
Source: BMC Genomics. 2016 Aug 5;17:552. doi: 10.1186/s12864-016-2860-6 (PMC4974748; doi:10.1186/s12864-016-2860-6)

# BUSCO Analysis

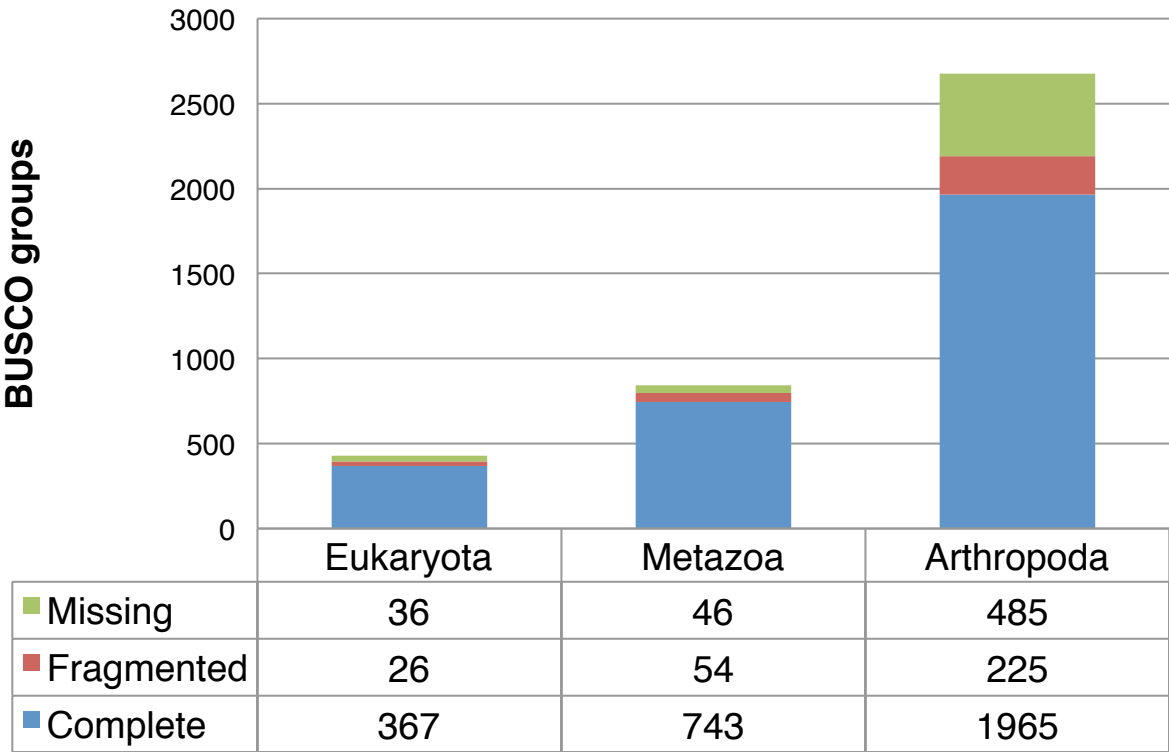

Supplement: Additional file 1: Figure S1. — Assessment of the Platynereis transcriptome assembly. To evaluate the completeness of our assembly, our gene models were compared with eukaryotic, metazoan, and arthropod universal single-copy orthologs using BUSCO. For the eukaryotic subset, the majority of eukaryote orthologs, 85 %, were completely assembled (blue), 6 % were partially assembled (crimson), and 8 % could not be identified in our assembly (green). The subsets of metazoan and arthropod orthologs have 88 and 73 % completeness, respectively. (PDF 30 kb) [file 12864_2016_2860_MOESM1_ESM.pdf]

**A**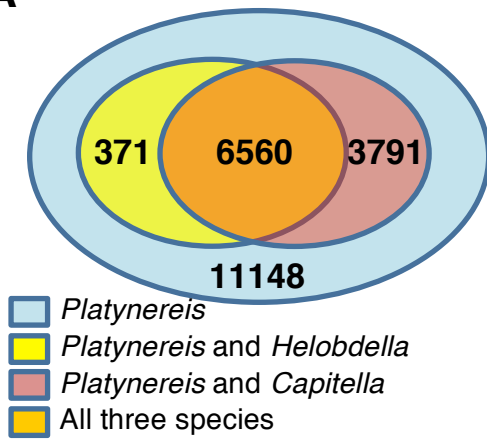**B**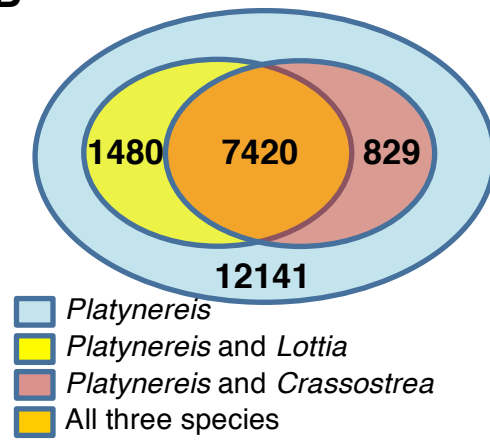**C**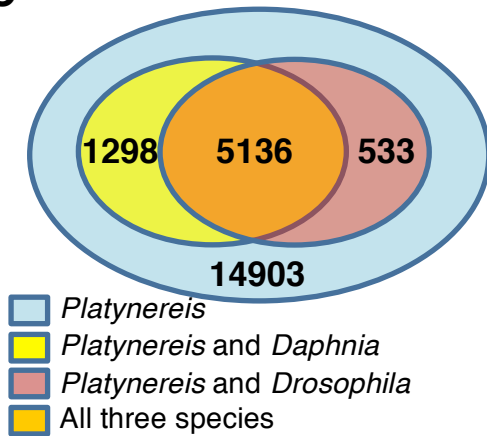**D**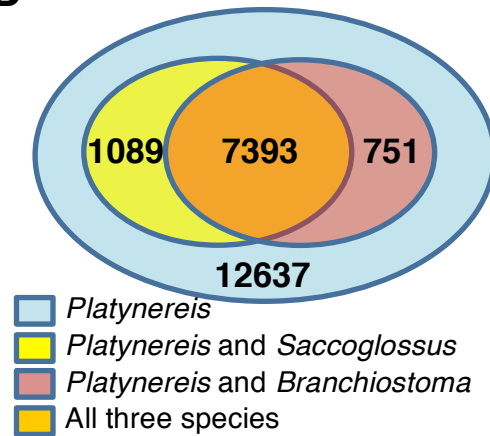**E**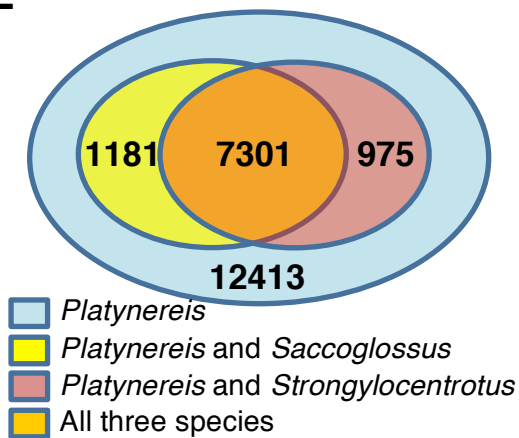**F**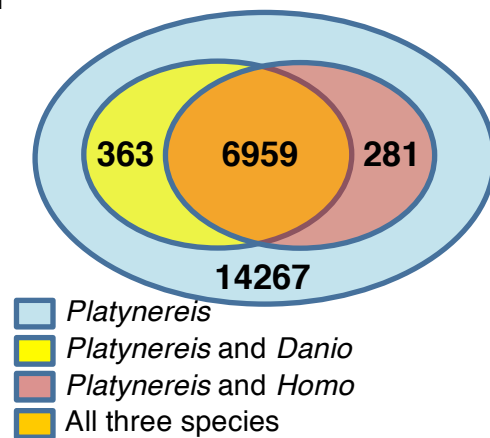**G**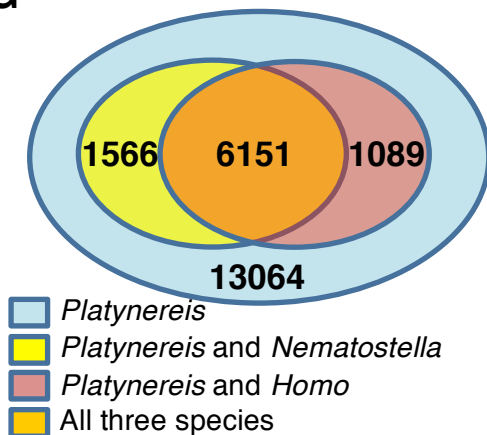

Supplement: Additional file 2: Figure S2. — Conservation of early Platynereis gene models in various metazoan species. Our early Platynereis transcriptome comprises of 21,870 gene models with ORFs >100 amino acids that are supported by the Swiss-Prot database, the OrthoMCL analysis, or the EMBL draft 1.90. To further understand the gene/protein evolution of these Platynereis gene models, we constructed orthologous groups among 18 selected species (see OrthoMCL analysis in Methods for details), and determined the number of shared genes between Platynereis and specific groups of species based on two stringent criteria: 1) shared genes must represent the best reciprocal blast hits between each species, and 2) the ORFs must share 50 % identity on the protein level. This figure shows Venn diagrams of the number of genes shared by the three selected species (orange), the number of genes shared between Platynereis and one of the species (yellow and crimson), and the number of genes in Platynereis only (blue). Each of the seven sets shown here contains Platynereis and two different species of similar phylogenetic distance. The 7 sets include: (A) Platynereis dumerilii, and two annelids, the leech Helobdella robusta and the bristle worm Capitella teleta. (B) Platynereis dumerilii, and two spiralian mollusks, the limpet Lottia gigantea and the pacific oyster Crassostrea gigas. (C) Platynereis dumerilii, and two ecdysozoans, the crustacean water flea Daphnia pulex and the insect fruit fly Drosophila melanogaster. (D) Platynereis dumerilii, and two invertebrate deuterostomes, the hemichordate Saccoglossus kowalevskii and the cephalochordate lancelet Branchiostoma floridae. (E) Platynereis dumerilii, and two deuterostome ambulacrarians, the acorn worm Saccoglossus kowalevskii and the echinoderm sea urchin Strongylocentrotus purpuratus. (F) Platynereis dumerilii, and two deuterostome vertebrates, the teleost zebrafish Danio rerio and the mammal human Homo sapiens. (G) Platynereis dumerilii, and two metazoans, the cnidarian [file 12864_2016_2860_MOESM2_ESM.pdf]

**A**

Color Key

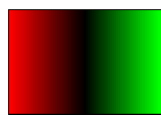

5 7 9  
Value

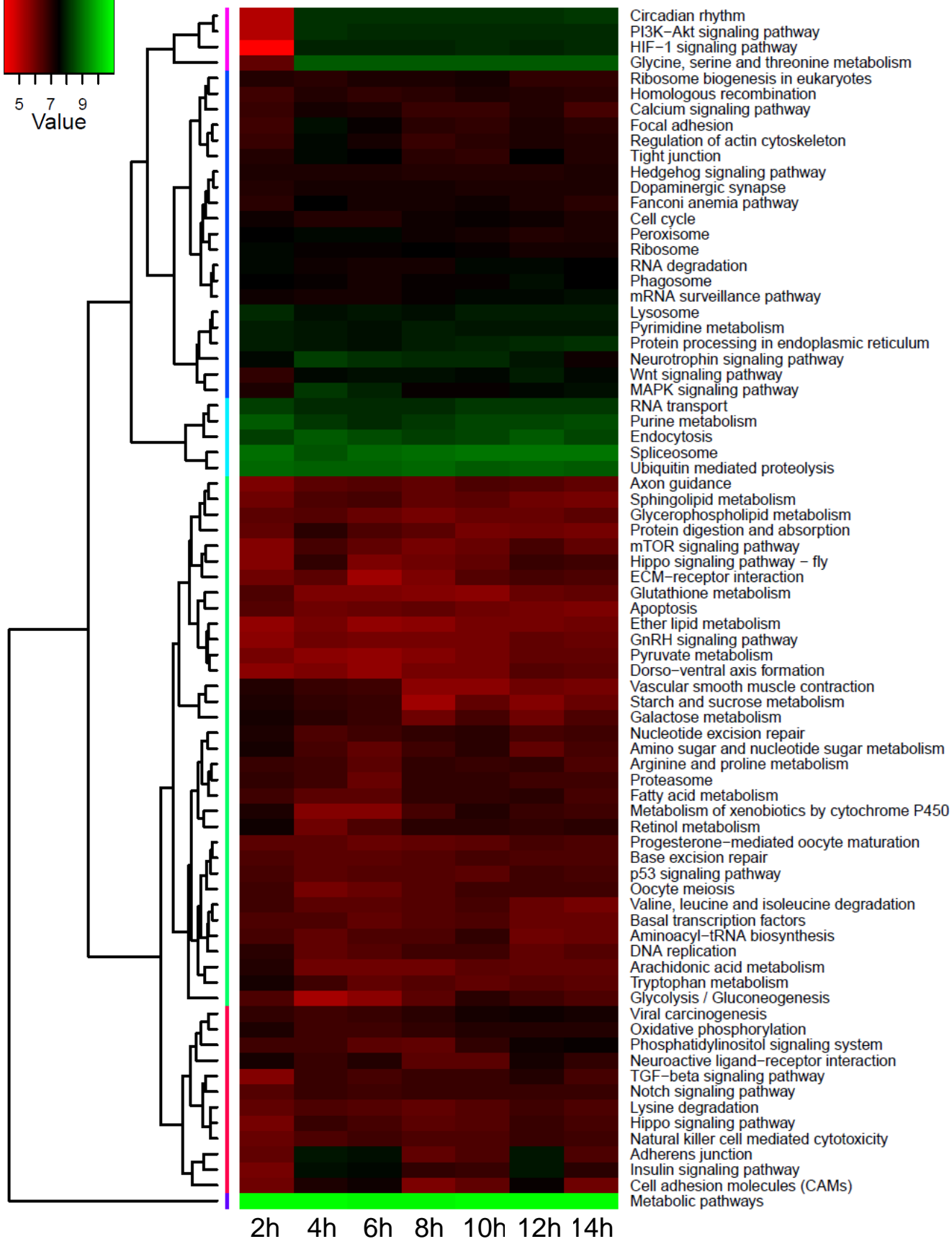

**B**

Color Key

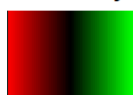

Value

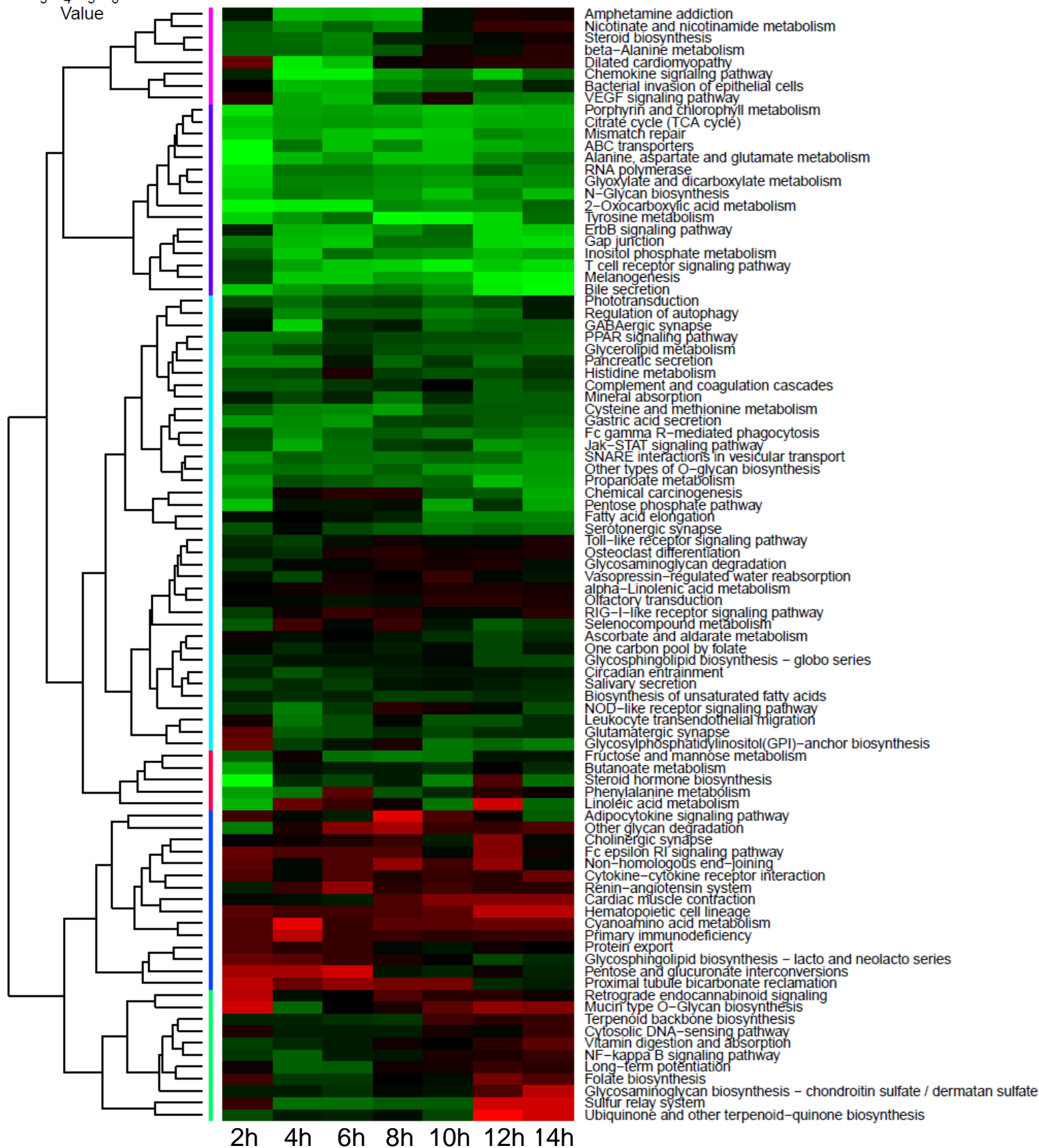

C

Color Key

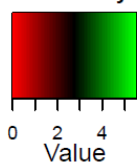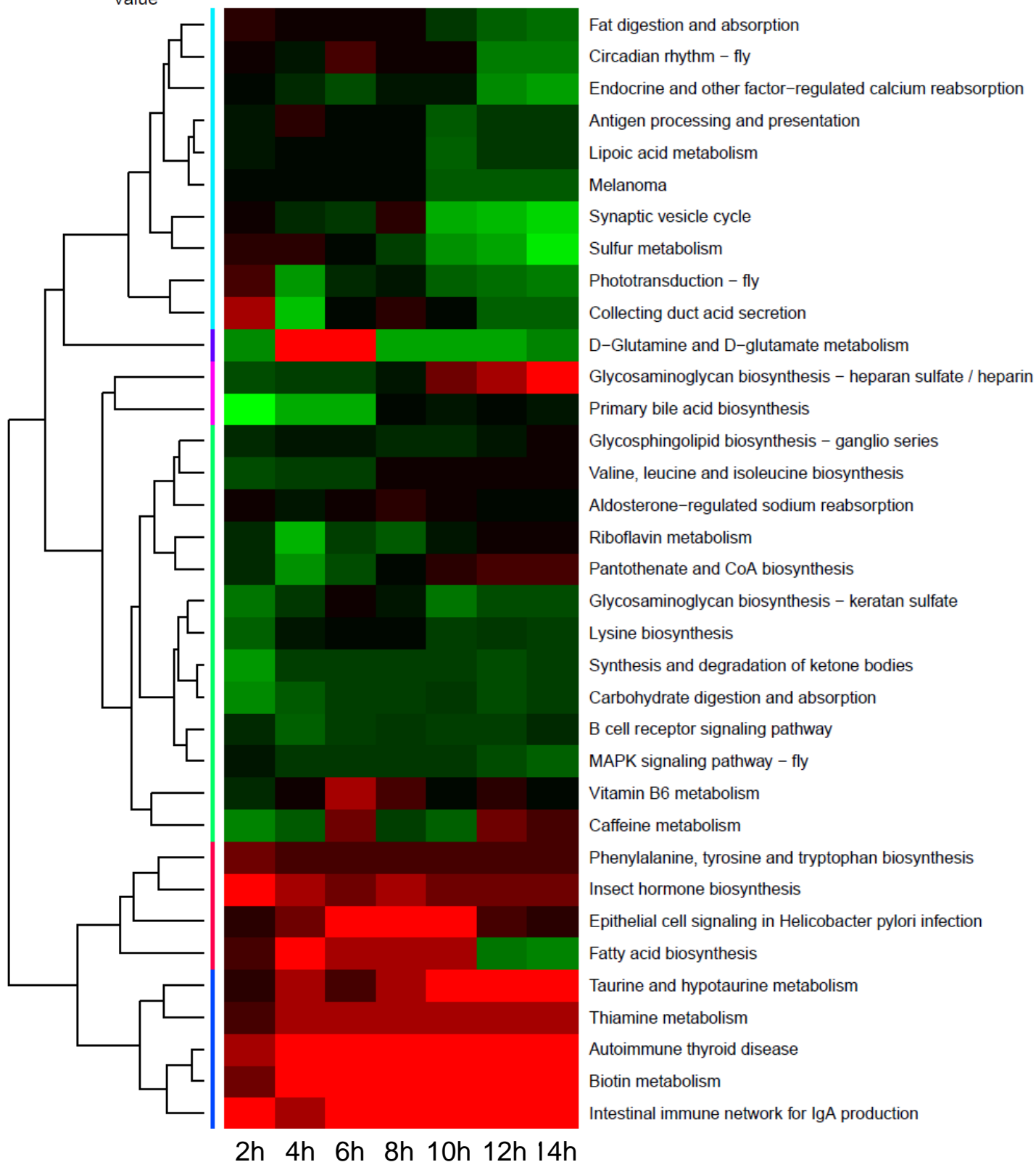

D

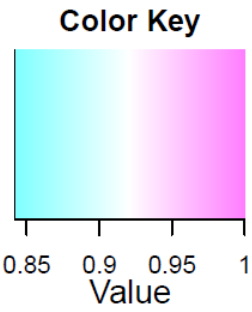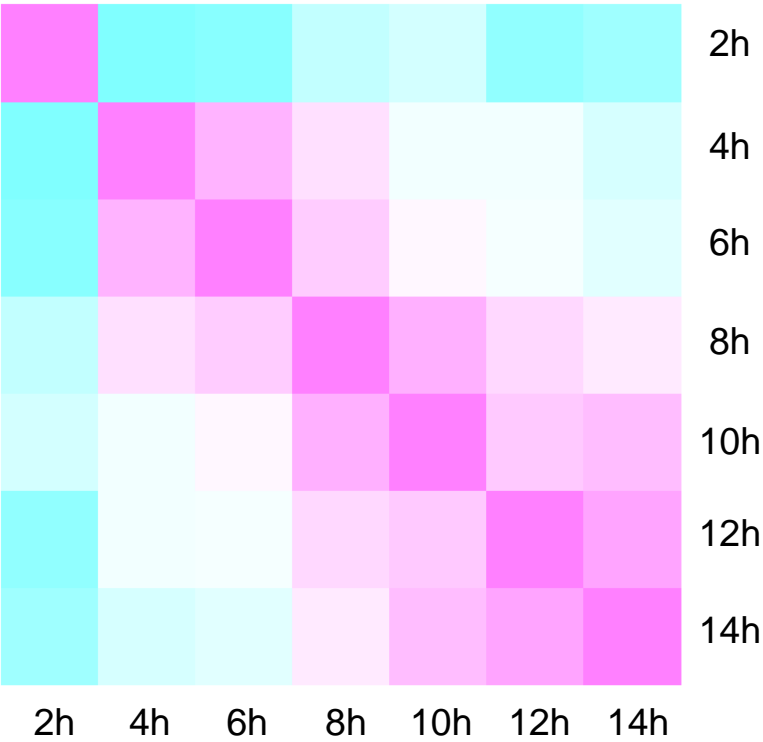

Supplement: Additional file 8: Figure S3. — KEGG enrichment analysis at each stage. The number of KEGG pathways associated with the top 5000 expressed genes at each stage was counted. Hierarchy clustering was used to classify these pathways and three distinct groups were observed: (A) High frequency (> 26), (B) Medium frequency (24 ~ 26), and (C) Low frequency (< 24). (D) All stages shared similar associated KEGG pathways (correlation coefficient >0.85). Adjacent stages have higher correlation with the exception of 2hpf, which is not similar to any other stage. (PDF 661 kb) [file 12864_2016_2860_MOESM8_ESM.pdf]

**A**

**Color Key**

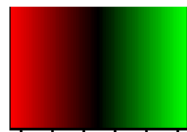

6 8 10  
Value

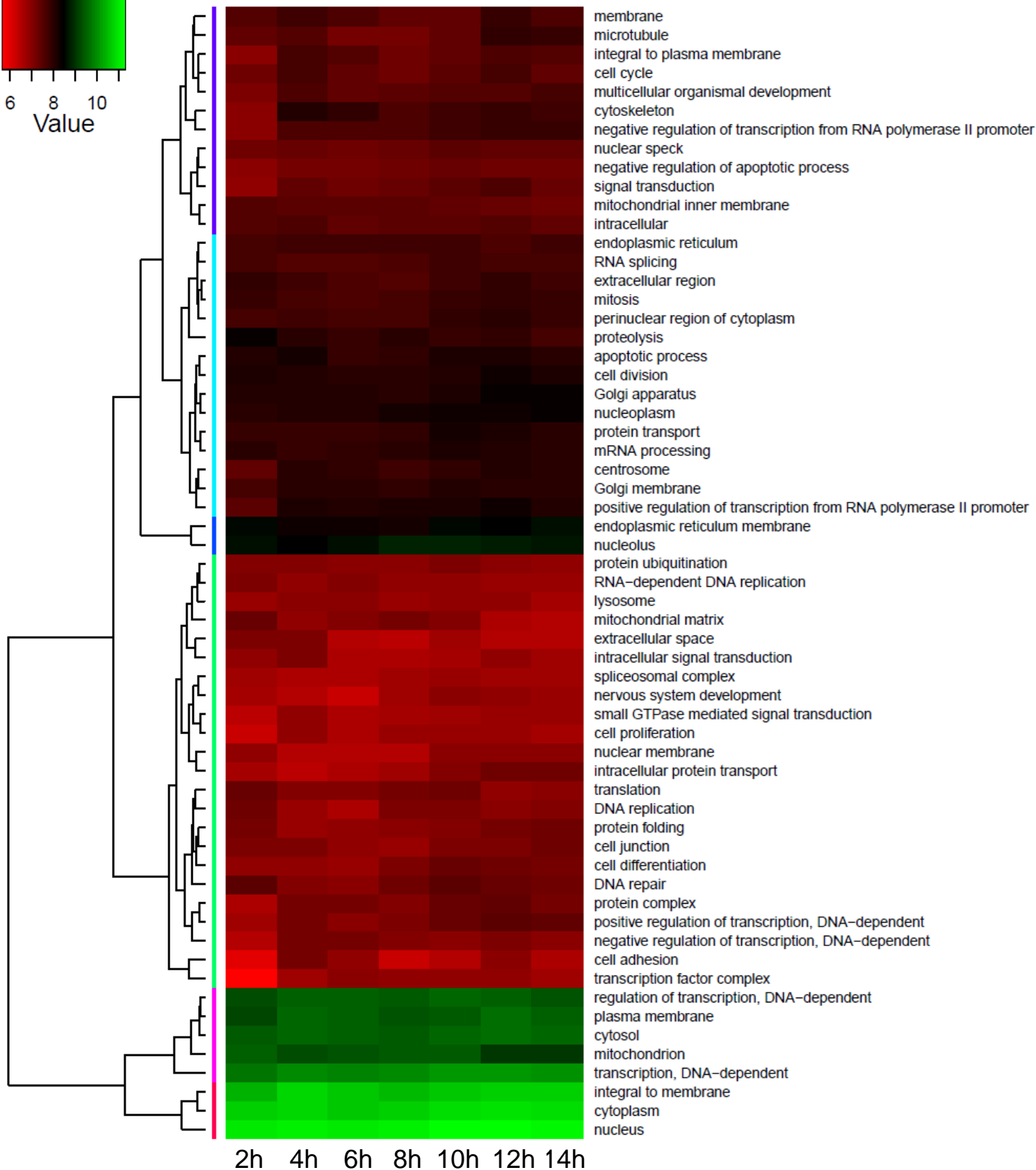

**B**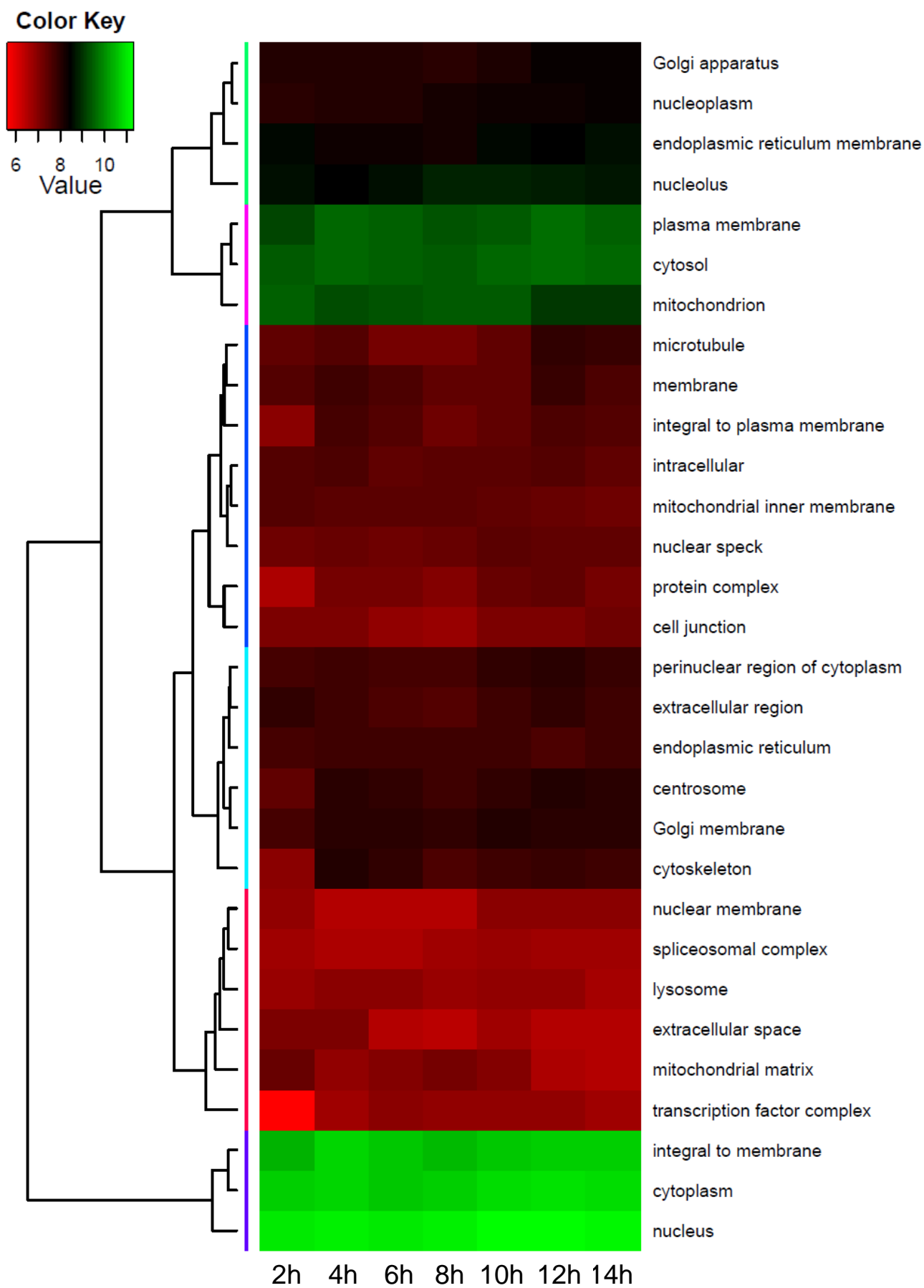

C

Color Key

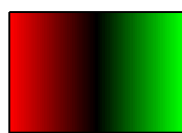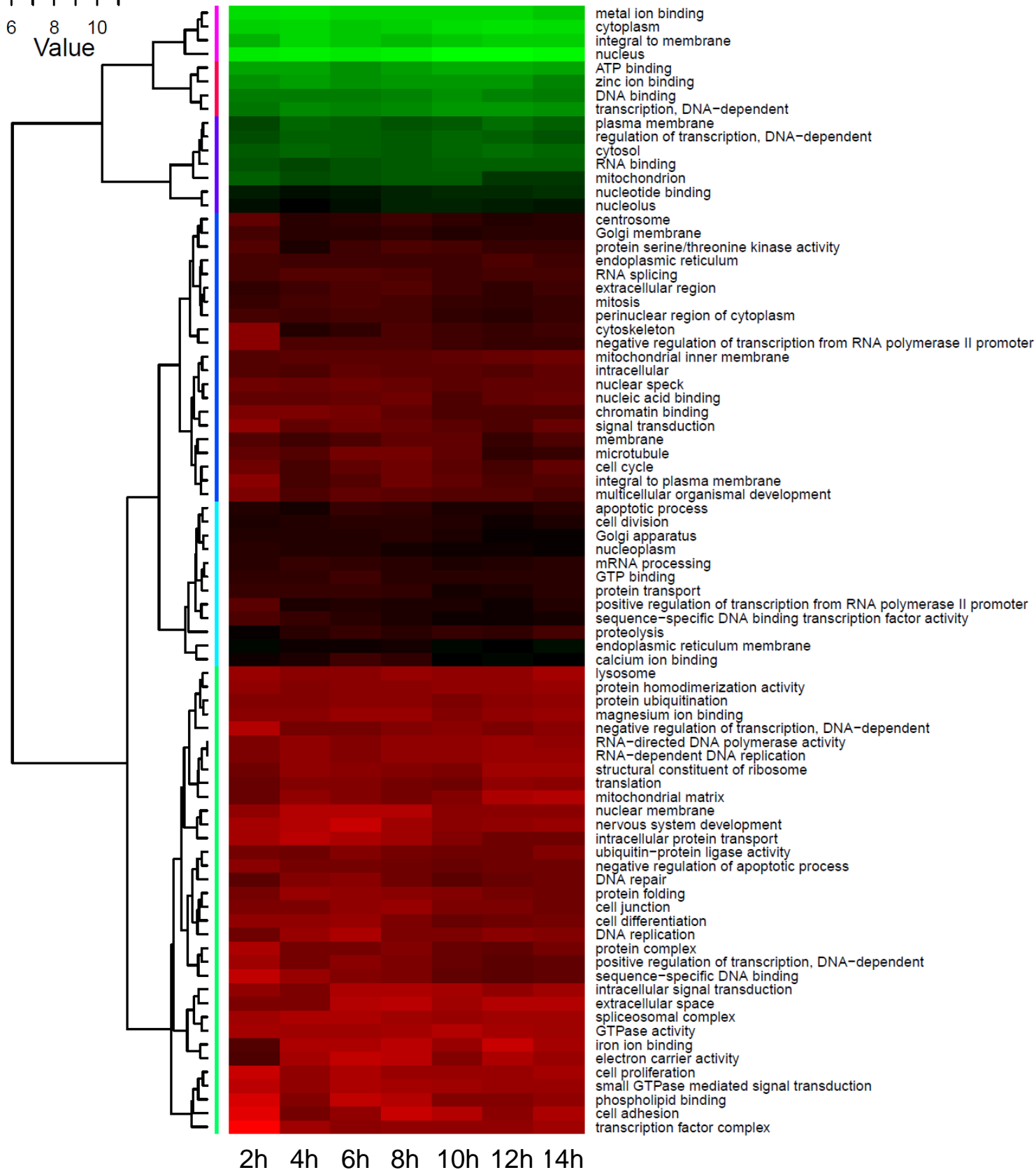

D

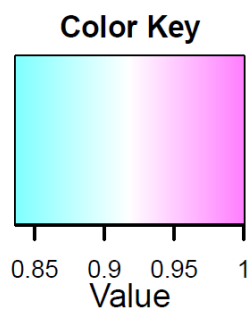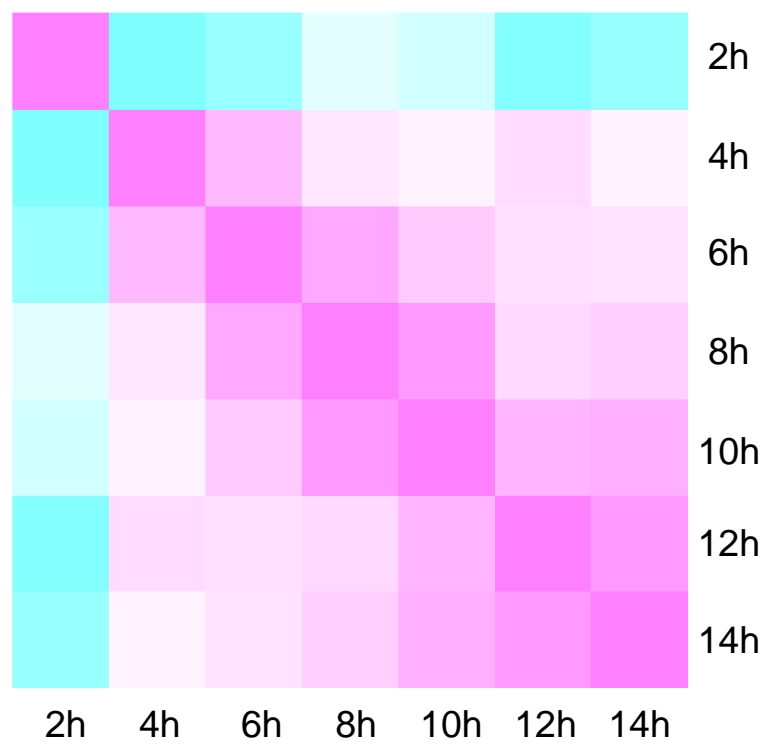

Supplement: Additional file 9: Figure S4. — Gene Ontology enrichment analysis at each stage. The top 5000 expressed genes at each stage were selected and their associated GO terms were shown by their namespaces: (A) Biological process, (B) Cellular component, and (C) Molecular function. (D) All stages show similar GO term enrichment patterns (correlation coefficient > 0.8). Similar to the KEGG pathways analysis, the 2hpf GO terms are less similar to the other six stages. (PDF 632 kb) [file 12864_2016_2860_MOESM9_ESM.pdf]
